# 4D printing of MXene hydrogels for high-efficiency pseudocapacitive energy storage

**DOI:** 10.1038/s41467-022-34583-0

**Published:** 2022-11-12

**Authors:** Ke Li, Juan Zhao, Ainur Zhussupbekova, Christopher E. Shuck, Lucia Hughes, Yueyao Dong, Sebastian Barwich, Sebastien Vaesen, Igor V. Shvets, Matthias Möbius, Wolfgang Schmitt, Yury Gogotsi, Valeria Nicolosi

**Affiliations:** 1 Centre for Research on Adaptive Nanostructures and Nanodevices (CRANN) & Advanced Materials and BioEngineering Research Centre (AMBER), Trinity College Dublin, Dublin, Dublin 2 Ireland; 2 School of Chemistry, Trinity College Dublin, Dublin, Dublin 2 Ireland; 3 School of Physics, Trinity College Dublin, Dublin, Dublin 2 Ireland; 4 A. J. Drexel Nanomaterials Institute and Department of Materials Science and Engineering, Drexel University, Philadelphia, PA 19104 USA

**Keywords:** Two-dimensional materials, Porous materials, Nanoscale materials, Supercapacitors

## Abstract

2D material hydrogels have recently sparked tremendous interest owing to their potential in diverse applications. However, research on the emerging 2D MXene hydrogels is still in its infancy. Herein, we show a universal 4D printing technology for manufacturing MXene hydrogels with customizable geometries, which suits a family of MXenes such as Nb_2_CT_*x*_, Ti_3_C_2_T_*x*_, and Mo_2_Ti_2_C_3_T_*x*_. The obtained MXene hydrogels offer 3D porous architectures, large specific surface areas, high electrical conductivities, and satisfying mechanical properties. Consequently, ultrahigh capacitance (3.32 F cm^−2^ (10 mV s^−1^) and 233 F g^−1^ (10 V s^−1^)) and mass loading/thickness-independent rate capabilities are achieved. The further 4D-printed Ti_3_C_2_T_*x*_ hydrogel micro-supercapacitors showcase great low-temperature tolerance (down to –20 °C) and deliver high energy and power densities up to 93 μWh cm^−2^ and 7 mW cm^−2^, respectively, surpassing most state-of-the-art devices. This work brings new insights into MXene hydrogel manufacturing and expands the range of their potential applications.

## Abstract (web summary)

2D MXene hydrogels are promising for diverse applications. Here, the authors report a universal 4D printing technology to manufacture MXene hydrogels with customizable geometry, high conductivity, and efficient pseudocapacitive energy storage ability.

## Introduction

The recent boom in portable electronics, hybrid/electric vehicles, and intermittent energy (e.g., sun and wind) harvesting highlights the need for efficient energy-storage systems^1,2^. Supercapacitors (SCs), as promising candidates, have stuck out because of their high power density and long cycle life, which enable fast charging and eliminate the need for replacement of energy-storage devices over the lifetime of equipment that they power. However, SCs suffer from low energy density which impedes their wide implementations; one feasible approach to bypass this is developing advanced electrode materials^3^.

Conductive hydrogels, particularly those based on conductive 2D materials (e.g., graphene and MXene), can be used as electrode materials with high energy and power densities^4,5^. They not only possess large surface areas and hydrophilic properties but also maintain the high electrical conductivity of 2D materials, allowing for electrochemical reactions, fast electrolyte ion transport and electron transfer even in thick electrodes^4^. 2D MXenes with a formula of M_n+1_X_n_T_*x*_ (M is an early transition metal, X is carbon and/or nitrogen, n is an integer between 1 and 4, and T_*x*_ represents surface functional groups) offer a large number of promising candidates for designing conductive 2D hydrogels owing to their large surface-area-to-volume ratios, high electrical conductivities (≥20,000 S cm^–1^), redox capable surface groups, and chemical/structural diversities^6–11^. To date, several MXene hydrogels have been developed by filtration^12^, or using metal ions^13^, graphene oxide^14^, or polymers (e.g., polyvinyl alcohol (PVA)^15^, polyacrylamide^16^, cellulose^17^, chitosan^18^, poly(acrylic acid)^19^, and poly(N-isopropylacrylamide)^20^) as crosslinkers, and have demonstrated some success. Nevertheless, research on MXene hydrogels is still in its infancy, and serious challenges remain. First, the previous reports all focused on Ti_3_C_2_T_*x*_; no other MXene hydrogels (beyond Ti_3_C_2_T_*x*_) have been reported. Second, the polymer crosslinkers employed were insulating, which lowered the electrical conductivity of MXene hydrogels and weakened their electrical/electrochemical performance. Third, the geometry of these MXene hydrogels strongly depends on the shape and size of molds, which is unlikely to meet the requirements of complexity and precision in many scenarios, especially in the context of the rapid development of portable electronics.

Additive manufacturing, or 3D printing, offers an efficient approach to realizing the precise, mold-free, and low-cost fabrication of complex objects by layer-by-layer deposition of material^21^. With the introduction of the fourth dimension of time, 4D printing (3D printing + time) emerged^22^. It not only inherits all merits of 3D printing but also allows the static objects created by 3D printing to intentionally change their shape, property, or functionality over time when exposed to specific external stimuli (e.g., heat, light, water, pH)^23^, endowing the printed objects with new features. However, no related works on MXene hydrogels were ever reported.

Herein, an advanced 4D printing technology is developed for scalable manufacturing of MXene hydrogels, with the conducting polymer poly(3,4-ethylenedioxythiophene):poly(styrene sulfonate) (PEDOT:PSS) as the crosslinker. Differing from the dissolvable MXene sol patterns produced by traditional 3D printing (Supplementary Table 1), in our 4D printing technology, crosslinked MXene hydrogels with enhanced mechanical strengths are obtained by employing a simple heat-stimulated self-assembly process. This strategy shows remarkable universality, which allows the fabrication of a series of MXene hydrogels including Nb_2_CT_*x*_, Ti_3_C_2_T_*x*_, and Mo_2_Ti_2_C_3_T_*x*_ hydrogels. Notably, these three kinds of MXenes possess different numbers of atomic layers (*n* = 1, 2, and 3) and different transition metals (Nb, Ti, Mo) on their surface, representing a large family of MXenes with diverse structures and properties. In addition, the geometries of the 4D-printed MXene hydrogels are precisely customizable: various complex architectures such as microlattice, rectangular hollow prism, Chinese knot, “CRANN” logo, and micro-supercapacitor (MSC) units are easily produced. Meanwhile, they all feature excellent hydrophilic properties, large specific surface areas, and high electrical conductivities. Consequently, the Ti_3_C_2_T_*x*_ hydrogel electrodes show a large areal capacitance of 3.32 F cm^−2^ at 10 mV s^−1^, an ultrahigh specific capacitance of 232.9 F g^−1^ at 10 V s^−1^, and mass loading/thickness-independent rate capabilities. Furthermore, the 4D-printed MXene hydrogel MSCs enable low-temperature operation, with high capacitance retentions of 90.6% at 0 °C and 82.2% at –20 °C. More importantly, the energy and power densities of our MSCs reach up to 92.88 μWh cm^−2^ and 6.96 mW cm^−2^, respectively, demonstrating their potential as efficient energy-storage devices.

## Results

### 4D printing of MXene hydrogels

**Fig. 1:**
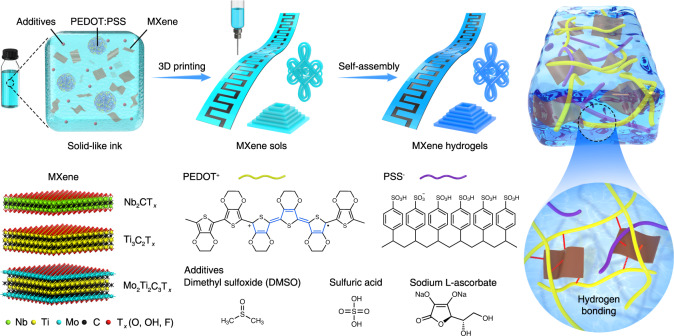
Schematic illustration of 4D printing of MXene hydrogels. The composite inks consisting of MXenes, PEDOT:PSS, and additives (DMSO, H_2_SO_4_, and sodium L-ascorbate) are 3D-printed into designed patterns first, followed by a self-assembly process, MXene sols transform into MXene hydrogels. Three kinds of MXenes, Nb_2_CT_*x*_, Ti_3_C_2_T_*x*_, and Mo_2_Ti_2_C_3_T_*x*_, are employed for demonstration of the universality and feasibility of this technology.

Figure 1 schematically represents the 4D printing methodology developed for manufacturing MXene hydrogels. A homogenous ink with the required rheological properties was formulated by mixing few-layer MXenes, PEDOT:PSS, and additives (dimethyl sulfoxide (DMSO), sulfuric acid (H_2_SO_4_), and sodium L-ascorbate). After 3D printing, various MXene sol patterns and architectures were obtained, which further transformed into MXene hydrogels via a heat-stimulated self-assembly process. Additives play a crucial role during the self-assembly process, as DMSO and H_2_SO_4_ simultaneously facilitate the self-assembly of MXene hydrogels, whilst sodium L-ascorbate is a reducing agent that protects MXenes from oxidation^24^.

**Fig. 2:**
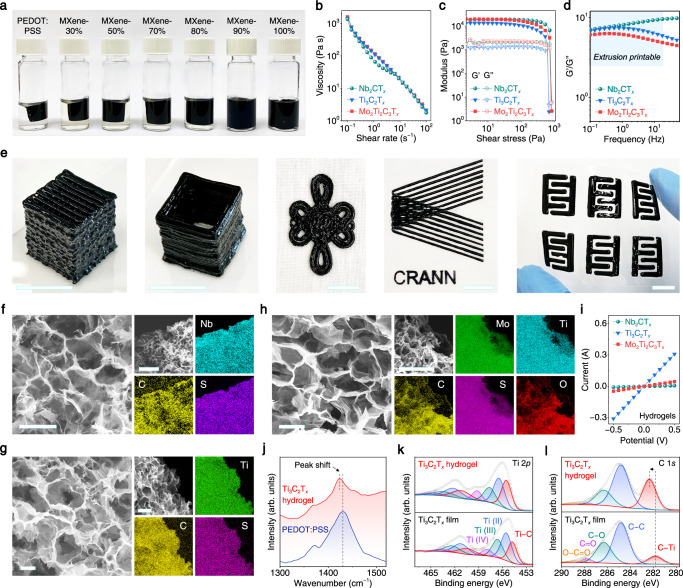
Characterizations of MXene inks and hydrogels. **a** Photographs of Ti_3_C_2_T_*x*_ hydrogels with different MXene contents prepared by self-assembly. **b** Viscosity as a function of shear rate for Nb_2_CT_*x*_, Ti_3_C_2_T_*x*_ and Mo_2_Ti_2_C_3_T_*x*_ inks. **c** Storage modulus (G′) and loss modulus (G′′) as a function of the shear stress for Nb_2_CT_*x*_, Ti_3_C_2_T_*x*_ and Mo_2_Ti_2_C_3_T_*x*_ inks. **d** Frequency dependency of the ratio of the G′ to G″ for Nb_2_CT_*x*_, Ti_3_C_2_T_*x*_ and Mo_2_Ti_2_C_3_T_*x*_ inks. **e** Photographs of 4D-printed MXene hydrogel architectures (from left to right): Ti_3_C_2_T_*x*_ hydrogel microlattice on glass slide, Ti_3_C_2_T_*x*_ hydrogel rectangular hollow prism on glass slide, Nb_2_CT_*x*_ hydrogel Chinese knot on cloth, Nb_2_CT_*x*_ hydrogel “CRANN” logo on PET film, flexible Mo_2_Ti_2_C_3_T_*x*_ hydrogel MSC units on PET film. All scale bars in **e** correspond to 1 cm. **f** SEM and energy-dispersive X-ray spectroscopy (EDX) mapping images of Nb_2_CT_*x*_ hydrogel. **g** SEM and EDX mapping images of Ti_3_C_2_T_*x*_ hydrogel. **h** SEM and EDX mapping images of Mo_2_Ti_2_C_3_T_*x*_ hydrogel. All scale bars in SEM images in **f**–**h** are 5 μm and all scale bars in EDX mapping images in **f**–**h** are 20 μm. **i** I−V curves of Nb_2_CT_*x*_, Ti_3_C_2_T_*x*_, and Mo_2_Ti_2_C_3_T_*x*_ hydrogels with a size of 10 × 2 × 2 mm. **j** Raman spectra of pure PEDOT:PSS film and 4D-printed Ti_3_C_2_T_*x*_ hydrogel. High resolution **k** Ti 2*p* and **l** C 1*s* XPS spectra of filtered Ti_3_C_2_T_*x*_ film and 4D-printed Ti_3_C_2_T_*x*_ hydrogel. Binding energies were all calibrated to the C 1*s* peak at 284.8 eV.

Nb_2_CT_*x*_, Ti_3_C_2_T_*x*_, and Mo_2_Ti_2_C_3_T_*x*_ MXenes were synthesized by etching their precursor MAX phases in hydrochloric acid-lithium fluoride (HCl-LiF) or hydrofluoric acid (HF) solutions (see Methods). The obtained MXenes all present characteristic peaks in the X-ray diffraction patterns (Supplementary Fig. 1) and contain ultrathin 2D flakes in the transmission electron microscopy (TEM) images (Supplementary Fig. 2), demonstrating their high quality. Since the self-assembly process is crucial for the formation of MXene hydrogels from MXene sols, this was first investigated on Ti_3_C_2_T_*x*_ MXene as an example, before moving to 3D printing. As shown in Fig. 2a, pure PEDOT:PSS hydrogel shows the most distinct volume shrinkage after self-assembly at 90 °C for 6 h. With the continuous introduction of Ti_3_C_2_T_*x*_ MXene, the volume contraction of hydrogels becomes progressively less prominent. Hydrogels could not be formed from pure MXene. The maximum load of Ti_3_C_2_T_*x*_ in hydrogels is 80 wt.%, and in this case, the produced Ti_3_C_2_T_*x*_ hydrogel could be readily transferred into another glass vial without breakage (Supplementary Fig. 3a). In addition, the shapes of Ti_3_C_2_T_*x*_ hydrogels (80 wt.%) are easily customizable: cones, hemispheres, cylinders, and fibers were all produced using different molds. More interestingly, the fiber-shaped Ti_3_C_2_T_*x*_ hydrogels show great flexibility, which has been demonstrated by patterning the letters “TCD” (Supplementary Fig. 3b). Thus, in the following experiments, the mass content of MXene in hydrogels was set to 80 wt.%. Apart from the MXene mass ratio, H_2_SO_4_ and DMSO also have crucial effects on the self-assembly process (Supplementary Figs. 4 and 5). In the absence of DMSO, the Ti_3_C_2_T_*x*_ hydrogels obtained in 0.1 M H_2_SO_4_ are rigid and fragile, and in 1 M H_2_SO_4_, hydrogels cannot even form (Supplementary Fig. 4a). While in absence of H_2_SO_4_, Ti_3_C_2_T_*x*_ hydrogels are soft and easily broken (Supplementary Fig. 4b), the volume fraction of DMSO also matters (Supplementary Fig. 5). Ultimately, the optimal formula for additives was determined to be 0.1 M H_2_SO_4_ plus 26 vol.% DMSO, and the molar ratio of the reducing agent sodium L-ascorbate to the metal atom in MXene was 1:1 (see Methods).

To enable 3D printing, the dispersions consisting of MXenes (Nb_2_CT_*x*_, Ti_3_C_2_T_*x*_, or Mo_2_Ti_2_C_3_T_*x*_), PDEOT:PSS, and additives in the above discussed proportions, were condensed by high-speed centrifugation, resulting in solid-like inks with MXene-PEDOT:PSS presenting a concentration of ~50 mg mL^–1^ (Supplementary Fig. 6). A rheological study was carried out to probe the suitability of these inks for 3D printing (Fig. 2b–d). As shown in Fig. 2b, the Nb_2_CT_*x*_, Ti_3_C_2_T_*x*_, and Mo_2_Ti_2_C_3_T_*x*_ inks all exhibit high apparent viscosities and shear-thinning non-Newtonian behavior, which ensures the continuous flow of inks through nozzles and the shape fidelity once the applied stress is released. Figure 2c depicts the storage modulus (G′) and loss modulus (G″) of the inks. The yield stress (crossover point between G′ and G″) values of Nb_2_CT_*x*_, Ti_3_C_2_T_*x*_, and Mo_2_Ti_2_C_3_T_*x*_ inks were measured to be 803, 664, and 778 Pa, respectively, which markedly exceed the requirement for 3D printing of MXenes (ca. 100 Pa)^25,26^. Below the yield stress (G′ > G″), the three inks behave as solids, and they flow under higher shear stress (G″ > G′), which allows for ink extrusion through nozzles. The frequency scan was also performed, as displayed in Supplementary Fig. 7, the three inks all possess higher G′ than G″ with G′ and G″ being frequency-independent throughout the measured frequency range. Moreover, the ink-processability-related parameter G′/G″ ratios of three inks fall within the range for MXene extrusion printing (from ca. 2.5 to 20) (Fig. 2d)^27^. This once again demonstrates the suitability of these inks for extrusion printing. Various MXene sol architectures were produced and are shown in Supplementary Movies 1–5. After a self-assembly process, the 3D-printed MXene sols further transformed into MXene hydrogels with great shape fidelity; a Ti_3_C_2_T_*x*_ hydrogel microlattice and a Ti_3_C_2_T_*x*_ hydrogel rectangular hollow prism on glass slides, a Nb_2_CT_*x*_ hydrogel Chinese knot on cloth, a Nb_2_CT_*x*_ hydrogel “CRANN” logo and flexible Mo_2_Ti_2_C_3_T_*x*_ hydrogel MSC units on polyethylene terephthalate (PET) films were fabricated with this technique to demonstrate its versatility and scalability (Fig. 2e, Supplementary Fig. 8). Additionally, treatment with concentrated H_2_SO_4_ boosted the mechanical properties of the 4D-printed hydrogels. Even after vigorous shaking, the treated Ti_3_C_2_T_*x*_ hydrogel microlattice and rectangular hollow prism retained their integrity without displaying breakage or deformation (Supplementary Movie 6, Supplementary Fig. 9). The enhanced mechanical strength will benefit MXene hydrogels in many applications. In contrast, the 3D-printed Ti_3_C_2_T_*x*_ sol microlattice broke into fragments after shaking (Supplementary Movie 7, Supplementary Fig. 10).

The microstructures of MXene hydrogels were examined with scanning electron microscopy (SEM). As displayed in Fig. 2f–h, the Nb_2_CT_*x*_, Ti_3_C_2_T_*x*_, and Mo_2_Ti_2_C_3_T_*x*_ hydrogels have porous structures with pore sizes ranging from less than a micrometer to several micrometers. TEM images also show the 3D framework of MXene hydrogel, and polymer chains sandwiched between MXene layers (Supplementary Fig. 11). N_2_ adsorption/desorption isotherms were recorded at 77 K to further investigate their Brunauer-Emmett-Teller (BET) specific surface areas (SSAs) and pore size distributions (Supplementary Fig. 12). The results confirmed the porosity of the MXene hydrogels materials, as indicated by their type II adsorption isotherms, typical of macroporous sheet-like adsorbents, but combined with hysteresis, typical of mesoporous materials (Type IV isotherms). While Mo_2_Ti_2_C_3_T_*x*_ and Nb_2_CT_*x*_ hydrogels present the expected narrow hysteresis loops, the Ti_3_C_2_T_*x*_ hydrogel presents a broader hysteresis which indicates the presence of slit-shaped mesopores^28^. The SSAs of Nb_2_CT_*x*_, Ti_3_C_2_T_*x*_, and Mo_2_Ti_2_C_3_T_*x*_ hydrogels were calculated to be 48.7, 21.8, and 21 m^2^ g^−1^, respectively, which are comparable to other porous MXene materials^29^. Notably, despite the porous structure inducing the formation of finite electron transport pathways, the electrical conductivities of our MXene hydrogels still reach 37, 1548, and 231 S m^−1^ for Nb_2_CT_*x*_, Ti_3_C_2_T_*x*_, and Mo_2_Ti_2_C_3_T_*x*_ hydrogels, respectively (Fig. 2i). This showcases the potential of these materials for applications in sensors, bioelectronics, electromagnetic interference shielding, electrochemical energy storage, etc. (Supplementary Table 2). The conductivity difference between these hydrogels should be ascribed to the intrinsic conductivity difference of the three MXenes (Supplementary Fig. 13).

### Probing self-assembly mechanism

To elucidate the self-assembly mechanism and the interactions between MXenes and PEDOT:PSS, Raman spectroscopy and X-ray photoelectron spectroscopy (XPS) were employed. In the Raman spectrum of the PEDOT:PSS film (Fig. 2j), the band at 1430 cm^−1^ arises from the C_α_=C_β_ stretching vibration of thiophene rings^30^, while it redshifts to 1422 cm^−1^ in the Ti_3_C_2_T_*x*_ hydrogel, suggesting the conformation change from benzene structure to quinoid structure and thus elongated conjugation lengths of PEDOT^+^ chains^31^. In addition, the narrower 1422 cm^−1^ band of the Ti_3_C_2_T_*x*_ hydrogel compared with the PEDOT:PSS film also confirms the increased crystallinity caused by the expansion and π–π stacking of the PEDOT^+^ chains^31^. In the high-resolution Ti 2*p* XPS spectra of Ti_3_C_2_T_*x*_ film and Ti_3_C_2_T_*x*_ hydrogel (Fig. 2k), all Ti_3_C_2_T_*x*_ hydrogel’s peaks shift to higher binding energy by about 1.0 eV, together with a slightly increased Ti−C^32^ contribution and somewhat decreased MXene surface groups (Supplementary Table 3). The peak corresponding to the C−Ti^33^ bond also shifts from 281.8 eV (Ti_3_C_2_T_*x*_ film) to 282.3 eV (Ti_3_C_2_T_*x*_ hydrogel) in the C 1*s* XPS spectra (Fig. 2l). Moreover, compared with Ti_3_C_2_T_*x*_ film, the C−Ti bond contribution in Ti_3_C_2_T_*x*_ hydrogel increases by three times (Supplementary Table 4), signifying the remarkably strengthened Ti−C interactions in the Ti_3_C_2_T_*x*_ hydrogel. Considerable variation also occurs in the S 2*p* XPS spectra, as depicted in Supplementary Fig. 14, where the ratio of PSS^−^ to PEDOT^+^ in pure PEDOT:PSS film is 2.2, while this value halves in Ti_3_C_2_T_*x*_ hydrogel (1.1), a strong indication of the prominent removal of PSS^−^ chains in Ti_3_C_2_T_*x*_ hydrogel.

Generally, PEDOT:PSS tends to form a core-shell structure with the hydrophilic PSS^−^ shell surrounding the hydrophobic PEDOT^+^ core^34^. PEDOT^+^ and PSS^−^ chains interact via electrostatic attraction, and the electrostatic repulsion between ionic PSS^−^ chains makes PEDOT:PSS hydrophilic and dispersible in water^35^. This feature enables the homogeneous blending of PEDOT:PSS with MXenes. Because of the electrostatic interaction and hydrogen bonding between MXene surface groups (−O, −OH, −F) and PEDOT:PSS^36^, the original electrostatic interactions between PEDOT^+^ and PSS^−^ are altered. Partial PEDOT^+^ chains attach to the negatively charged MXene surface and oxidize from benzene to quinoid configuration^37^. Once H_2_SO_4_ is introduced, the abundant H^+^ ions further protonate PSS^−^ and weaken the electrostatic attraction between PEDOT^+^ and PSS^−^ chains^38^, as well as the repulsion force between PEDOT:PSS micelles^31^. As a result, more coiled PEDOT^+^ chains expand to linear structures with elongated conjugation lengths, and more PSS^−^ chains are removed from PEDOT:PSS and dissolved in the acidic solution. These exposed PEDOT^+^ chains not only interact with each other to form physical crosslinks through π–π stacking and hydrophobic interaction^34^ but also interact with MXene sheets via electrostatic attraction and hydrogen bonding and bridge them into 3D networks (Figs. 1, 2f–h and Supplementary Fig. 11). Meanwhile, the strengthened Ti–C bonds and reduced MXene surface groups are observed (Supplementary Tables 3 and 4), owing to the robust interactions between MXene and PEDOT^+^. Polar DMSO is a secondary dopant^39^ that promotes the removal of PSS^−^ from PEDOT:PSS, like H_2_SO_4_, and they synergistically contribute to the self-assembly process (Supplementary Figs. 4 and 5). The heating treatment is indispensable during self-assembly, which accelerates these processes and strengthens the interactions, facilitating the generation of robust MXene hydrogels within a short time.

### High-rate electrochemical performance of 4D-printed MXene hydrogel electrodes

**Fig. 3:**
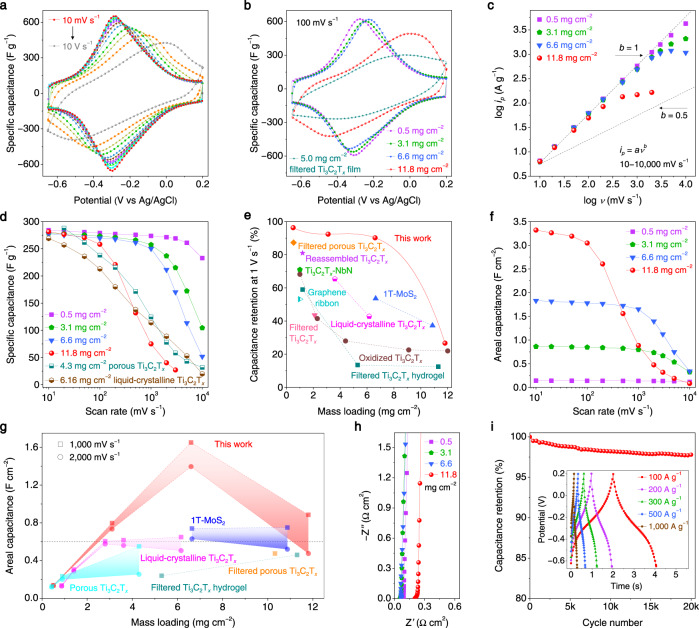
Electrochemical performance of 4D-printed Ti_3_C_2_T_*x*_ hydrogel electrodes. **a** CV curves of Ti_3_C_2_T_*x*_ hydrogel (0.5 mg cm^−2^) at scan rates of 10, 20, 50, 100, 200, 500, 1000, 2000, 3000, 5000, and 10,000 mV s^−1^. **b** CV curves of Ti_3_C_2_T_*x*_ hydrogels with different mass loadings at a scan rate of 100 mV s^−1^. **c** Determination of the slope, *b*-value, for Ti_3_C_2_T_*x*_ hydrogels with different mass loadings. **d** Rate performance of Ti_3_C_2_T_*x*_ hydrogels at scan rates from 10 to 10,000 mV s^−1^. The porous Ti_3_C_2_T_*x*_ (4.3 mg cm^−2^)^12^ and liquid-crystalline Ti_3_C_2_T_*x*_ (6.16 mg cm^−2^)^41^ are plotted for comparison. **e** Capacitance retentions of Ti_3_C_2_T_*x*_ hydrogels with different mass loadings at 1 V s^−1^. Lots of high-rate electrodes are listed for comparison, including filtered porous Ti_3_C_2_T_*x*_^43^, reassembled Ti_3_C_2_T_*x*_^50^, Ti_3_C_2_T_*x*_-NbN^51^, graphene ribbon^52^, filtered Ti_3_C_2_T_*x*_^53^, liquid-crystalline Ti_3_C_2_T_*x*_^41^, 1T-MoS_2_^42^, oxidized Ti_3_C_2_T_*x*_^54^, and filtered Ti_3_C_2_T_*x*_ hydrogel^12^. **f** Areal capacitance of Ti_3_C_2_T_*x*_ hydrogels with different mass loadings at scan rates from 10 to 10,000 mV s^−1^. **g** Comparison of the areal capacitance of Ti_3_C_2_T_*x*_ hydrogels with benchmark electrodes at scan rates of 1 and 2 V s^−1^. These electrodes are porous Ti_3_C_2_T_*x*_^12^, liquid-crystalline Ti_3_C_2_T_*x*_^41^, 1 T−MoS_2_^42^, filtered Ti_3_C_2_T_*x*_ hydrogel^12^, and filtered porous Ti_3_C_2_T_*x*_^43^. **h** EIS plots of Ti_3_C_2_T_*x*_ hydrogels with different mass loadings taken at 0.2 V. **i** Long-term stability of Ti_3_C_2_T_*x*_ hydrogel performed by cycling at 100 mV s^−1^. The inset depicts galvanostatic charge-discharge (GCD) profiles of Ti_3_C_2_T_*x*_ hydrogel (1.0 mg cm^−2^) at ultrahigh current densities of 100, 200, 300, 500, and 1000 A g^−1^, respectively. The GCD curves of this Ti_3_C_2_T_*x*_ hydrogel at low current densities of 1, 2, 3, 5, and 10 A g^−1^ are shown in Supplementary Fig. 20.

To demonstrate the feasibility of 4D-printed MXene hydrogels for electrochemical energy storage, we chose Ti_3_C_2_T_*x*_ hydrogel as a model and investigated its electrochemical performance as supercapacitor electrode in Swagelok cell (Supplementary Fig. 15) (PEDOT:PSS hydrogel and Ti_3_C_2_T_*x*_ film were also tested for comparison, Supplementary Fig. 16). As shown in Fig. 3a, Ti_3_C_2_T_*x*_ hydrogel (0.5 mg cm^−2^) shows distinct redox peaks at approximately −0.3 V versus Ag/AgCl reference electrode at a scan rate of 10 mV s^−1^, and negligible distortion of cyclic voltammetry (CV) curves is observed even at 10 V s^−1^, indicating an ultrafast and highly reversible pseudocapacitive energy-storage process. This is also supported by the small cathodic and anodic peak potentials separation (less than 100 mV for scan rates up to 1 V s^−1^) (Supplementary Fig. 17). In addition, differing from the loss of pseudocapacitive characteristics in the filtered Ti_3_C_2_T_*x*_ electrode (5.0 mg cm^−2^) at a medium scan rate of 100 mV s^−1^ (Fig. 3b), redox peaks of Ti_3_C_2_T_*x*_ hydrogels remain independent of mass loading/thickness from 0.5 mg cm^−2^ (0.12 mm) to 6.6 mg cm^−2^ (1.5 mm), and are evident even in a 2.9 mm-thick hydrogel with a commercially relevant mass loading of 11.8 mg cm^−2^. This suggests the substantially enhanced ion-transport properties and mass loading/thickness-independent behavior of Ti_3_C_2_T_*x*_ hydrogels.

To interpret the capacitive performance of Ti_3_C_2_T_*x*_ hydrogels, charge-storage kinetic analysis was carried out (Fig. 3c). The peak-current density (*i*_*p*_) and scan rate (*v*) from CV curves follow the relationship: *i*_*p*_ = *av*^*b*^, where *a* and *b* are variables. *b*-value is an indicator of the charge-storage kinetics, which is determined from the slope of the plot of log *i*_*p*_ versus log *v*^40^. Apparently, the *b*-value remains close to 1 in a wide scan-rate range from 10 to 2000 mV s^−1^ for Ti_3_C_2_T_*x*_ hydrogels with mass loadings up to 6.6 mg cm^−2^, revealing that their charge-storage kinetics is surface-controlled. The diffusion-controlled mechanism becomes prominent when the mass loading of Ti_3_C_2_T_*x*_ hydrogel reaches 11.8 mg cm^−2^, which should be ascribed to the long ion diffusion distance in thick electrodes.

Figure 3d shows the rate performance of Ti_3_C_2_T_*x*_ hydrogels with different mass loadings/thicknesses. It is worth noting that, at an ultrahigh scan rate of 10 V s^−1^, the 0.12 mm-thick hydrogel (0.5 mg cm^−2^) delivers a record high specific capacitance of 232.9 F g^−1^, retaining 82% capacitance at 10 mV s^−1^. For thicker and higher mass loading hydrogels (0.7 mm/3.1 mg cm^−2^, 1.5 mm/6.6 mg cm^−2^), their rate performance decays are also minor at scan rates up to 1 V s^−1^, superior to the previously reported 180 μm-thick porous Ti_3_C_2_T_*x*_ (4.3 mg cm^−2^)^12^ and 320 μm-thick liquid-crystalline Ti_3_C_2_T_*x*_ (6.16 mg cm^−2^)^41^. The mass loading/thickness-independent rate performance of Ti_3_C_2_T_*x*_ hydrogels is further demonstrated in Fig. 3e. At a fixed high scan rate of 1 V s^−1^, ultrahigh capacitance retentions of 96.3%, 92.4%, and 90.2% are achieved for Ti_3_C_2_T_*x*_ hydrogels with 0.5, 3.1, and 6.6 mg cm^−2^ mass loadings, respectively, pronouncedly surpassing most state-of-the-art high-rate electrodes (Supplementary Table 5).

Ti_3_C_2_T_*x*_ hydrogel electrodes also show remarkable areal capacitance. As displayed in Fig. 3f, the maximum areal capacitance of Ti_3_C_2_T_*x*_ hydrogel (11.8 mg cm^−2^) reaches 3.32 F cm^−2^ at 10 mV s^−1^, greater than most advanced MXene electrodes (Supplementary Table 5). Meanwhile, the areal capacitance grows linearly with mass loadings from 0.5 to 11.8 mg cm^−2^ at scan rates up to 100 mV s^−1^ (Supplementary Fig. 18). At a high scan rate of 2 V s^−1^, this linear relationship is still maintained within a mass loading range of 0.5–6.6 mg cm^−2^ (Fig. 3g), indicating fast ion diffusion and high-efficiency energy-storage in Ti_3_C_2_T_*x*_ hydrogels. In commercial applications, the typical areal capacitance of electrode is 0.6 F cm^−2^ (horizontal dash line in Fig. 3g)^42^, and to date, only the liquid-crystalline Ti_3_C_2_T_*x*_^41^ and 1T-MoS_2_^42^ electrodes have achieved this value at 1 V s^−1^. Hydrogel electrodes not only meet the commercial requirements but also possess two times higher areal capacitance than liquid-crystalline Ti_3_C_2_T_*x*_ and 1T-MoS_2_ electrodes at both 1 and 2 V s^−1^ under similar mass loadings (6.16–6.6 mg cm^−2^). At the maximum mass loading of 11.8 mg cm^−2^, the areal capacitance of our Ti_3_C_2_T_*x*_ hydrogel at 1 V s^−1^ retains 0.89 F cm^−2^, which is higher than 1T-MoS_2_^42^, filtered porous Ti_3_C_2_T_*x*_^43^, and filtered Ti_3_C_2_T_*x*_ hydrogel^12^ electrodes.

Electrochemical impedance spectroscopy (EIS) measurements were conducted to offer deeper insights into the ion transport and charge transfer behavior in MXene hydrogels. As displayed in Fig. 3h, all Ti_3_C_2_T_*x*_ hydrogel electrodes, including the 2.9 mm-thick one (11.8 mg cm^−2^), show low series resistances (less than 0.2 Ω cm^2^). However, the ion diffusion resistance (Warburg impedance) between the MXene hydrogels and filtered MXene films is quite different (Supplementary Fig. 19). Unlike the filtered Ti_3_C_2_T_*x*_ electrode with a clear 45° slope in the mid-frequency region, plots of all Ti_3_C_2_T_*x*_ hydrogels are nearly vertical at all the measured frequencies, certainly demonstrating the faster ion diffusion in hydrogel electrodes, which is vital for mass loading/thickness-independent performance. Moreover, the Ti_3_C_2_T_*x*_ hydrogel is highly stable, remaining 97.8% capacitance after cycling for 20,000 times at a scan rate of 100 mV s^−1^ (Fig. 3i). All these results show a potential of MXene hydrogels to enable ultrahigh charge-discharge rates and long-term cycling stability within thick electrodes with high mass loading.

### Charge-storage performance of 4D-printed MSCs

**Fig. 4:**
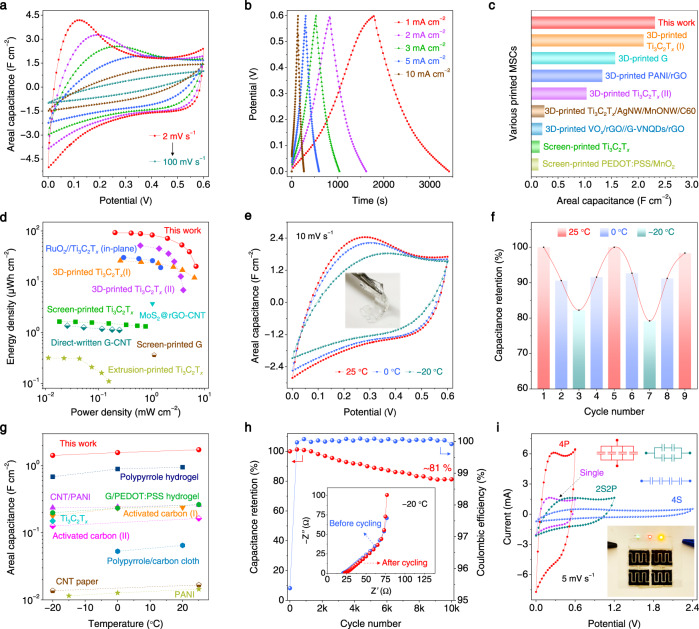
Electrochemical performance of 4D-printed Ti_3_C_2_T_*x*_ hydrogel MSCs. **a** CV curves of 4D-printed Ti_3_C_2_T_*x*_ hydrogel MSC at scan rates of 2, 5, 10, 20, 50, and 100 mV s^−1^. **b** GCD curves of 4D-printed Ti_3_C_2_T_*x*_ hydrogel MSC at current densities of 1, 2, 3, 5, and 10 mA cm^−2^. **c** Comparison of areal capacitance of 4D-printed Ti_3_C_2_T_*x*_ hydrogel MSC with other printed MSCs. **d** Ragone plots of 4D-printed Ti_3_C_2_T_*x*_ hydrogel MSC and other high-performance MSCs. **e** CV curves of 4D-printed Ti_3_C_2_T_*x*_ hydrogel MSC at a scan rate of 10 mV s^−1^ at 25 °C, 0, and −20 °C. Inset is the PVA-EG-H_2_SO_4_ gel electrolyte at −20 °C, showing great transparency and flexibility. **f** Capacitance retention of 4D-printed Ti_3_C_2_T_*x*_ hydrogel MSC during cooling/heating cycles. **g** Comparison of areal capacitance of 4D-printed Ti_3_C_2_T_*x*_ hydrogel MSC (10 mV s^−1^) with other devices at low temperatures. **h** Cycling performance of 4D-printed Ti_3_C_2_T_*x*_ hydrogel MSC at a current density of 30 mA cm^−1^ at −20 °C, inset shows the EIS data of this MSC before and after 10,000 cycles at −20 °C. **i** CV curves of single 4D-printed Ti_3_C_2_T_*x*_ hydrogel MSC, four MSCs in series (4 S), four MSCs in parallel (4 P), and two in series and in parallel (2S2P), at a scan rate of 5 mV s^−1^. Insert shows the photograph of a 4 S tandem device powering three LED indicators, demonstrating the feasibility of our MSCs for practical applications.

To investigate the suitability of MXene hydrogels for practical energy-storage applications, a symmetric Ti_3_C_2_T_*x*_ hydrogel MSC (mass loading ~35 mg cm^−2^) was 4D-printed and tested in a polyvinyl alcohol-ethylene glycol-sulfuric acid (PVA-EG-H_2_SO_4_) gel electrolyte (Supplementary Fig. 21). Notably, the highly conductive Ti_3_C_2_T_*x*_ hydrogel (1548 S m^−1^) does not require current collector. The CV curves of the 4D-printed Ti_3_C_2_T_*x*_ hydrogel MSC at scan rates from 2 to 100 mV s^−1^ at room temperature (25 °C) are shown in Fig. 4a. The stable operating voltage window of this MSC reaches 0.6 V, together with a pair of redox peaks observed at around 0 V, which is an indicator of the pseudocapacitive behavior and agrees with the nonlinear GCD curves (Fig. 4b). According to the CV data, the areal capacitance of the 4D-printed Ti_3_C_2_T_*x*_ hydrogel MSC reaches 2.31 F cm^−2^ at a scan rate of 2 mV s^−1^ and retains 0.53 F cm^−2^ when the scan rate is increased by 50-fold (100 mV s^−1^) (Supplementary Fig. 22). This performance surpasses most of printed MSCs (Fig. 4c, Supplementary Table 6), such as 3D-printed Ti_3_C_2_T_*x*_ MSC (2.1 F cm^−2^ at 1.7 mA cm^−2^)^26^, 3D-printed graphene MSC (1.57 F cm^−2^ at 2 mA cm^−2^)^44^, and screen-printed Ti_3_C_2_T_*x*_ MSC (0.158 F cm^−2^ at 0.08 mA cm^−2^)^45^. The areal energy and power densities of the 4D-printed Ti_3_C_2_T_*x*_ hydrogel MSC are also extraordinary. As displayed in Fig. 4d, the maximum areal energy and power densities of our MSC achieve 92.88 μWh cm^−2^ and 6.96 mW cm^−2^, respectively. These values are greater than most reported MSCs (Supplementary Table 7), for instance, 3D-printed Ti_3_C_2_T_*x*_ MSCs (51.7 μWh cm^−2^, 4 mW cm^−2^)^46^, screen-printed Ti_3_C_2_T_*x*_ MSC (1.64 μWh cm^−2^, 0.778 mW cm^−2^)^45^, and direct-written graphene-CNT MSC (1.36 μWh cm^−2^, 0.25 mW cm^−2^)^47^. It is worth mentioning that, upon optimizing the configuration of the devices (e.g., mass loading and finger electrode gap), the electrochemical performance of our MSC could be further enhanced^45^.

Low-temperature adaptability is crucial for electrochemical energy-storage devices in practical applications, but accomplishing this goal remains challenging due to the reduced mobility of electrolyte near/below their freezing points^48,49^. Importantly, the PVA-EG-H_2_SO_4_ gel electrolyte we used is not frozen but shows great transparency and mechanical flexibility even at −20 °C (Fig. 4e inset), differing from the icy PVA-H_2_SO_4_ electrolyte with a white color (Supplementary Fig. 23). This can be attributed to the anti-freezing feature of EG towards water and the abundant hydrogen bonds between EG molecules and PVA chains^49^. More importantly, the ionic conductivity of PVA-EG-H_2_SO_4_ maintains as high as 27.5 mS cm^−1^ at −20 °C (Supplementary Fig. 24, Supplementary Table 8), which facilitates the rapid electrolyte ions diffusion at subzero temperatures. The highly conductive and porous Ti_3_C_2_T_*x*_ hydrogel with enhanced electrolyte accessibility also potentially alleviates the adverse effects of low temperatures. Consequently, excellent low-temperature tolerance is achieved for the 4D-printed Ti_3_C_2_T_*x*_ hydrogel MSC, retaining 90.6% capacitance at 0 °C and 82.2% capacitance at −20 °C, in comparison with that at 25 °C (Fig. 4e). These values are significantly higher than other reports (Supplementary Fig. 25). Even after cooling and heating for several cycles between −20, 0, and 25 °C, the capacitance of our MSC recovers, demonstrating its remarkable low-temperature reversibility (Fig. 4f). Moreover, at –20 °C, the areal capacitance of 4D-printed Ti_3_C_2_T_*x*_ hydrogel MSC reaches 1.42 F cm^−2^ (10 mV s^−1^), superior to most high-performance low-temperature tolerant supercapacitors ever reported (Fig. 4g) (Supplementary Table 9).

To further demonstrate the potential of 4D-printed Ti_3_C_2_T_*x*_ hydrogel MSC for practical application, particularly in cold environments, a cycling test at –20 °C was conducted. As shown in Fig. 4h, our MSC retains ~81% of initial capacitance and ~100% Coulombic efficiency after 10,000 cycles. The excellent electrochemical durability is also demonstrated by the nearly unaltered EIS curves before and after cycling (Fig. 4h insert). In addition, our MSC units can be arbitrarily connected in series and/or in parallel to meet the specific energy/power requirements in real scenarios (Fig. 4i). For instance, a four in series (4 S) tandem device can readily power three LED bulbs (Fig. 4i insert).

## Discussion

We have developed an advanced 4D printing technology for the efficient fabrication of MXene hydrogels. This strategy is universal in its application to a family of MXenes with different atomic layers and transition metal types (e.g., Nb_2_CT_*x*_, Ti_3_C_2_T_*x*_, and Mo_2_Ti_2_C_3_T_*x*_) and a series of MXene hydrogels with complex and precise architectures on various substrates were successfully manufactured. These included a Nb_2_CT_*x*_ hydrogel Chinese knot on cloth, a Nb_2_CT_*x*_ hydrogel “CRANN” logo on PET film, a Ti_3_C_2_T_*x*_ hydrogel microlattice and a Ti_3_C_2_T_*x*_ hydrogel rectangular hollow prism on glass slides, and Mo_2_Ti_2_C_3_T_*x*_ hydrogel micro-supercapacitor units on PET film. The obtained MXene hydrogels all present 3D porous structures, large specific surface areas, and high electrical conductivities. As a result, highly efficient pseudocapacitive energy storage could be achieved, including ultrahigh capacitances, excellent mass loading/thickness-independent rate capabilities, great low-temperature tolerances, and high areal energy/power densities (92.88 μWh cm^−2^, 6.96 mW cm^−2^). This work offers new insights into the manufacture of MXene hydrogels and will advance the applications of MXenes and conductive hydrogels in electrochemical energy storage and conversion, sensors, bioelectronics, electromagnetic interference shielding, water purification, and other technologies.

## Methods

### Synthesis of Mo_2_Ti_2_AlC_3_ MAX phase

To synthesize the Mo_2_Ti_2_AlC_3_ MAX precursor, Mo (250 mesh, Alfa Aesar, 99.9%), Ti (325 mesh, Alfa Aesar, 99.5%), Al (325 mesh, Alfa Aesar, 99.5%), and graphite (325 mesh, Alfa Aesar, 99%) powders were used, with an atomic ratio of 2:2:1.3:2.7 (50 g total). The powder mixtures were then mixed in a 2:1 ball:powder ratio with 5 mm alumina balls, and ball milled at 60 rpm for 24 h. Afterward, high-temperature sintering reactions at 1600 °C for 4 h in a Carbolite furnace were conducted, with a heating and cooling rate of 3 °C min^−1^, and 200 cm^3^ min^−1^ flow of ultrahigh purity Ar (99.999%). After cooling down, the porous compact product was milled using a TiN-coated milling bit and sieved through a 400-mesh sieve, producing powders with a particle size <38 μm. The powders were then added into 9 M HCl (Sigma–Aldrich, 37%) to dissolve any residual metals or intermetallics and washed with deionized water by filtration until neutral. All experiments on this study were conducted on a single batch of MAX to eliminate any artifacts from variation between MAX synthesis batches.

### Synthesis of few-layer Nb_2_CT_*x*_

One gram of Nb_2_AlC MAX (400 mesh, 98%, Jilin 11 Technology, Co. Ltd, China) was first added slowly (over 10 min) into a mixture solution consisting of 2 g of LiF (Alfa Aesar, 97%) and 20 mL of 12 M HCl and stirred at 60 °C for 96 h, yielding multilayer Nb_2_CT_*x*_, which was further washed with 1 M HCl and deionized water by centrifugation (Heraeus Multifuge X1 Centrifuge, Thermo Fisher Scientific, USA) at 3005 × *g* (4000 rpm) until the supernatant became neutral. To produce few-layer Nb_2_CT_*x*_, the as-synthesized multilayer Nb_2_CT_*x*_ was dispersed in 20 mL of 5 wt. % (diluted) tetramethylammonium hydroxide (TMAOH) solution (Sigma–Aldrich, 25 wt.%) and stirred at room temperature for 12 h. Afterward, the mixture was centrifuged directly at 4696 × *g* (5000 rpm) for 20 min, and then washed with 40 mL of deionized water by centrifugation at 15,777 × *g* (12,000 rpm) to remove excess TMAOH. The obtained precipitate was redispersed in deionized water and sonicated for 1 h in an ice bath with N_2_ bubbling. After another centrifugation at 3005 × *g* (4000 rpm) for 1 h, few-layer Nb_2_CT_*x*_ dispersion with a dark color was collected.

### Synthesis of few-layer Ti_3_C_2_T_*x*_

One gram of Ti_3_AlC_2_ MAX (400 mesh, Carbon, Ukraine) was first added slowly (over 10 min) into a mixture solution consisting of 1 g of LiF and 20 mL of 9 M HCl and stirred at 35 °C for 48 h, yielding multilayer Ti_3_C_2_T_*x*_, which as further washed with deionized water by centrifugation at 3005 × *g* until the supernatant became neutral. To produce few-layer Ti_3_C_2_T_*x*_, the as-synthesized multilayer Ti_3_C_2_T_*x*_ was redispersed in deionized water and sonicated for 1 h in an ice bath with N_2_ bubbling. After another centrifugation at 3005 × *g* for 1 h, few-layer Ti_3_C_2_T_*x*_ dispersion with a dark color was collected.

### Synthesis of few-layer Mo_2_Ti_2_C_3_T_*x*_

One gram of Mo_2_Ti_2_AlC_3_ MAX was first added slowly (over 10 min) into 20 mL of HF (Acros Organics, 48–50 wt.%) and stirred at 55 °C for 96 h, yielding multilayer Mo_2_Ti_2_C_3_T_*x*_, which was further washed with deionized water by centrifugation at 3005 × *g* until the supernatant became neutral. To produce few-layer Mo_2_Ti_2_C_3_T_*x*_, the as-synthesized multilayer Mo_2_Ti_2_C_3_T_*x*_ was dispersed in 20 mL of 5 wt. % TMAOH solution and stirred at 35 °C for 12 h. Afterward, the mixture was centrifuged directly at 4696 × *g* for 20 min, and then washed with 40 mL of deionized water by centrifugation at 15,777 × g to remove excess TMAOH. The obtained precipitate was redispersed in deionized water and sonicated for 1 h in an ice bath with N_2_ bubbling. After another centrifugation at 3005 × *g* for 1 h, few-layer Mo_2_Ti_2_C_3_T_*x*_ dispersion with a dark color was collected.

### Preparation of MXene hydrogels by self-assembly method in molds

Typically, 3.2 mL of few-layer Ti_3_C_2_T_*x*_ MXene suspension (20 mg mL^−1^) and 1.52 mL of PEDOT:PSS suspension (PH 1000, Clevios^TM^, ~10.5 mg mL^−1^) were first mixed into a homogenous dispersion by stirring for 30 min and sonication for 10 min. Another mixture solution containing 2.6 mL of DMSO (Sigma–Aldrich, 99.9%, anhydrous), 0.33 mL of 3 M H_2_SO_4_ (Honeywell, 95–97%), 1.15 mL of 1 M sodium L-ascorbate (Acros Organics, 99%), and 1.2 mL of deionized water was added in dropwise and kept stirring for 30 min. Afterward, the obtained black mixture with a concentration of 8 mg mL^−1^ was transferred into sealed molds (e.g., glass vials and capillary tubes) with different shapes and sizes and heated at 90 °C for 6 h in oven, resulting in black Ti_3_C_2_T_*x*_ MXene hydrogels (MXene mass content: 80 wt.%). The as-prepared hydrogels were further immersed in 3 M and 18.4 M H_2_SO_4_ for 24 h to enhance their mechanical strength and washed with deionized water to remove any impurities. Notably, when optimizing the self-assembly conditions, the mass content of MXene in MXene-PEDOT:PSS mixture varied from 0, 30, 50, 70, 80, 90, to 100 wt.%, the volume proportion of DMSO to the whole dispersion (include MXene suspension, PEDOT:PSS suspension, additives and deionized water) varied from 0, 13, 26, to 50 vol.%, and the concentration of H_2_SO_4_ in the whole dispersion was set as 0.1 M or 1 M. The molar ratio of the reducing agent sodium L-ascorbate to metal atom in MXene was fixed at 1:1.

### 4D printing of MXene hydrogels

A black mixture dispersion of MXene (Nb_2_CT_*x*_, Ti_3_C_2_T_*x*_, or Mo_2_Ti_2_C_3_T_*x*_), PEDOT:PSS, and additives (0.1 M H_2_SO_4_, 26 vol.% DMSO and specific amount sodium L-ascorbate) was first condensed by centrifugation at 10,956 × *g* (10,000 rpm), resulting in solid-like inks with MXene-PEDOT:PSS presenting a concentration of ~50 mg mL^−1^. The inks were then loaded into a 5 mL syringe with a stainless-steel nozzle (0.26 mm) and being 3D-printed into different architectures via a commercial 3D printer (nano3Dprint). The printing speed was set as 1000 mm min^−1^. After 3D printing, the obtained MXene sols were sealed in a glass container and heated at 90 °C for 6 h in oven, to accomplish the transformation of MXene sols into MXene hydrogels. These MXene hydrogels were further immersed in 3 M and 18.4 M H_2_SO_4_ for 24 h to enhance their mechanical strength (3 M and 12 M H_2_SO_4_ employed when PET substrates were utilized to avoid their dissolution). Afterward, the obtained MXene hydrogels were immersed in deionized water and washed several times to remove any impurities. The mass contents of MXenes in three hydrogels are 80 wt.% and the solid contents of three MXene hydrogels are ~4.2 wt.%.

### Preparation of low-temperature tolerant polymer gel electrolyte

2.67 mL of EG (Hach) was first mixed with 5.33 mL of deionized water, then 1 g of PVA (Aldrich, Mw 89,000–98,000, 99%, hydrolyzed) was introduced and stirred at 85 °C for 4 h until dissolved. After cooling down completely, another solution consisting of 3 g of 18.4 M H_2_SO_4_, 0.67 mL of EG, and 1.33 mL of deionized water was added in slowly, and stirred for another hour, yielding the PVA-EG-H_2_SO_4_ gel electrolyte.

### Fabrication of micro-supercapacitors

4D-printed MXene hydrogel MSC (Supplementary Fig. 21, mass loading ~35 mg cm^−2^, size 2.2 × 1.7 × 0.4 cm (L × W × H)) were vacuum-dried (~0.5 mbar) at room temperature first, then two silver wires were connected separately to the two electrodes by conductive silver paint. After completely drying, nail polish was further coated on the top of Ag paint to protect it from potential exposure to electrolytes. Afterward, a layer of PVA-EG-H_2_SO_4_ gel electrolyte (1.8 mL) was cast, and the obtained MSCs were stored in a vacuum desiccator for 10 min to facilitate the electrolyte permeation. It is worth noting that, benefiting from the high electrical conductivity of MXene hydrogels, no extra metal current collectors were employed. The size of devices shown in Fig. 4i is 2.2 × 1.7 × 0.05 cm, and 0.22 mL of gel electrolyte was employed.

### Material characterization

The morphology of MXene hydrogels was observed using SEM (Zeiss ULTRA plus) and TEM (FEI Titan 80–300, 300 kV). Surface chemical information of the samples was analyzed by an Omicron Multiprobe XPS instrument equipped with a monochromatic Al Kα X-ray source (h*ν* = 1486.6 eV). Raman spectra were recorded with a Horiba Labram Aramis Raman spectrometer at 633 nm (grating 1800 gr/mm, 10% of laser power). XRD patterns were obtained using a powder diffractometer (Bruker D8 Discover) with Cu Kα1 radiation. The N_2_ adsorption/desorption measurements were performed on Quantachrome Autosorb-iQ. Before measurement, the samples (vacuum-dried at room temperature) were further vacuum-dried at 150 °C for 10 h. The rheological properties of the inks were investigated using an Anton Paar MCR 302e rheometer using a 50 mm plate-plate geometry. The upper plate was a roughened plate (PP50/S) to lessen the effects of slip and a solvent trap was used to minimize the effects of evaporation.

### Electrochemical measurements

All electrochemical tests were performed on a Biologic VMP300 potentiostat. For the three-electrode test, plastic Swagelok cells (PFA-420-3) were used (Supplementary Fig. 15). MXene hydrogels on glassy carbon, overcapacitive activated carbon (YP-50, Kuraray, Japan), Ag/AgCl in 3.5 M KCl, and 3 M H_2_SO_4_ (0.5 mL) were used as working electrode, counter electrode, reference electrode, and electrolyte respectively. For the MSC device test, vacuum-dried MXene hydrogels and PVA-EG-H_2_SO_4_ were the electrode materials and gel electrolyte, respectively. The room-temperature electrochemical tests were done in ambient conditions with an average temperature of 25 °C. The low-temperature electrochemical tests were conducted in a lab fridge. EIS was measured ranging from 10 mHz to 1000 kHz under a potential amplitude of 10 mV.

### Calculations for the electrochemical tests

*b*-value calculation: the highest current *i* during discharging, or the current at the highest peak, was recorded as a function of the scan rate *ν* in the range of 10–10,000 mV s^−1^. By fitting log *i* as a function of log *ν*, a linear curve was obtained with its slope as the *b* value.

The specific capacitance (F g^−1^) of a single electrode was calculated based on CV curves:

1 $${C}_{{sp}}=\frac{\int i\cdot t}{m\times V},$$

where *i* is the current, *t* is the discharging time, *m* is the mass of entire electrode, *V* is the voltage window of CV scan.

The areal capacitance (F cm^−2^) of a single electrode was calculated as:

2 $${C}_{s}={M}_{e}\times {C}_{{sp}},$$

where *M*_*e*_ is the mass loading of entire electrode.

As the GCD curves of electrodes are not linear, the capacity (mAh g^−1^) of a single electrode, instead of capacitance, was calculated from the GCD curve as:

3 $$C=\frac{i\times \triangle t}{3.6\times m},$$

where *i* is the current, Δ*t* is the discharging time, and *m* is the mass of entire electrode.

For the two-electrode device (MSC), the areal capacitance (F cm^−2^) was evaluated based on CV curves:

4 $${C}_{s,{{{{{{\mathrm{MSC}}}}}}}}=\frac{\int i\cdot t}{{S}_{{{{{{{\mathrm{MSC}}}}}}}}\times {V}_{{{{{{{\mathrm{MSC}}}}}}}}},$$

where *i* is the current, *t* is the discharging time, *S*_*MSC*_ is the entire area of MSC (including both the electrodes and gap), *V*_*MSC*_ is the voltage window of MSC.

The areal energy density (mWh cm^−2^) and average power density (mW cm^−2^) of MSC was calculated based on the discharge scan of GCD curves:

5 $${E}_{{{{{{{\mathrm{MSC}}}}}}}}=\frac{i}{{S}_{{{{{{{\mathrm{MSC}}}}}}}}}\times \left({\int }_{0}^{{t}_{{{{{{{\mathrm{MAX}}}}}}}}}{V}_{{{{{{{\mathrm{MSC}}}}}}}}\cdot {dt}\right),$$

and

6 $${P}_{{{{{{{\mathrm{MSC}}}}}}}}=\frac{{E}_{{{{{{{\mathrm{MSC}}}}}}}}}{\triangle t},$$

where *i* is the current, *S*_*MSC*_ is the entire area of MSC (including both the electrodes and gap), *t*_*MAX*_ is the maximum discharge time, *V*_*MSC*_ is the voltage window of MSC, Δ*t* is the discharging time.

Ionic conductivity (mS cm^−1^) of PVA-EG-H_2_SO_4_ gel electrolyte was calculated according to this formula:

7 $$\sigma=\frac{L}{R\times S},$$

where *L* is the thickness of electrolyte, *R* is the resistance of electrolyte, and *S* is the cross-section area of electrolyte.

## Supplementary information

Supplementary Information

Peer Review File

Description of Additional Supplementary Files

Supplementary Movie 1

Supplementary Movie 2

Supplementary Movie 3

Supplementary Movie 4

Supplementary Movie 5

Supplementary Movie 6

Supplementary Movie 7

## Data Availability

The data that support the findings of this study are available from the corresponding authors upon reasonable request. Source data are provided with this paper.

## Source data

Scource data
