# Supplementary material for: 4D printing of MXene hydrogels for high-efficiency pseudocapacitive energy storage
Source: Nat Commun. 2022 Nov 12;13:6884. doi: 10.1038/s41467-022-34583-0 (PMC9653467; doi:10.1038/s41467-022-34583-0)
Supplement: Supplementary file 1 — Supplementary Information [file 41467_2022_34583_MOESM1_ESM.pdf]

Supplementary Information

4D Printing of MXene Hydrogels for High-Efficiency Pseudocapacitive Energy Storage

Ke Li[1,2*], Juan Zhao[1,2], Ainur Zhussupbekova[2,3], Christopher E. Shuck[4], Lucia Hughes[1,2], Yueyao Dong[2], Sebastian Barwich[3], Sebastien Vaesen[1,2], Igor V. Shvets[3], Matthias Möbius[3], Wolfgang Schmitt[1,2], Yury Gogotsi[4*], Valeria Nicolosi[1,2*]

[1]Centre for Research on Adaptive Nanostructures and Nanodevices (CRANN) & Advanced Materials and BioEngineering Research Centre (AMBER), Trinity College Dublin, Dublin, Dublin 2, Ireland.

[2]School of Chemistry, Trinity College Dublin, Dublin, Dublin 2, Ireland.

[3]School of Physics, Trinity College Dublin, Dublin, Dublin 2, Ireland.

[4]A. J. Drexel Nanomaterials Institute and Department of Materials Science and Engineering, Drexel University, Philadelphia, PA 19104, USA.

* Corresponding author: like@tcd.ie (K.L.); gogotsi@drexel.edu (Y.G.); nicolov@tcd.ie (V.N.)

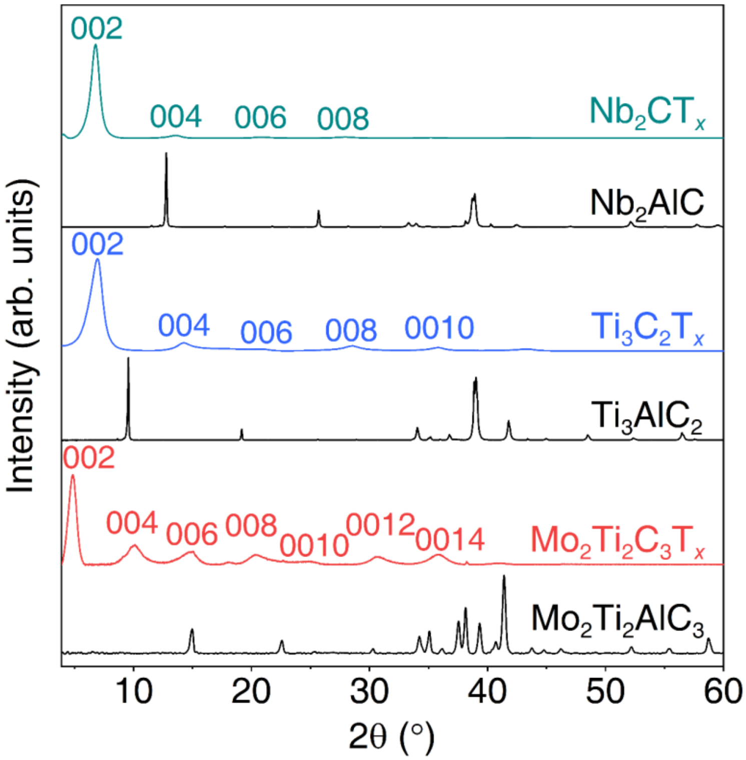

Supplementary Figure 1 | Phase characterization of MXenes and MAX phases. X-ray diffraction patterns of Nb_2CT_x, $Ti_3C_2T_x$, and $Mo_2Ti_2C_3T_x$ MXenes and their MAX phase precursors. The three MXenes all show clear characteristic diffraction peaks and match well with previous reports[1, 2, 3], demonstrating their high quality and purity.

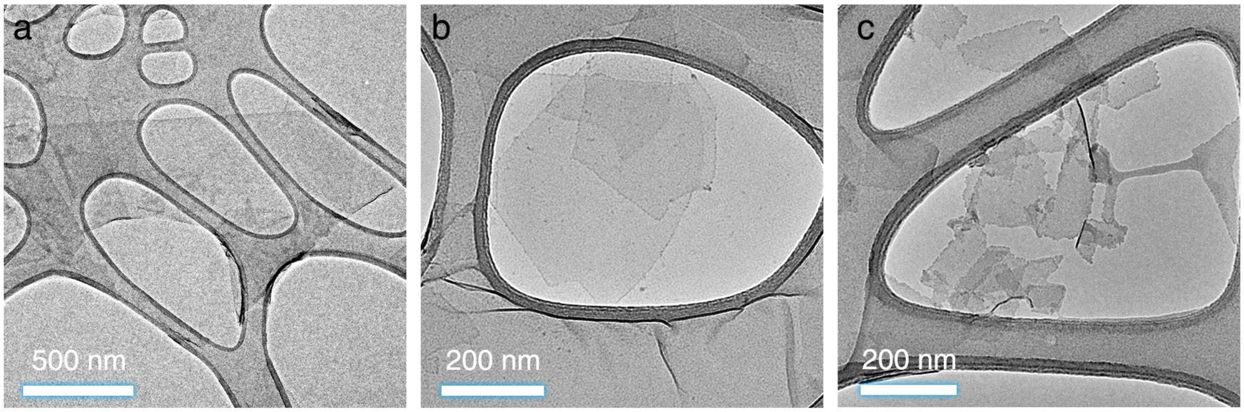

Supplementary Figure 2 | Morphology characterization of MXenes. TEM images of (a) Nb_2CT_x, (b) $Ti_3C_2T_x$, and (c) $Mo_2Ti_2C_3T_x$ MXenes. They all show ultrathin 2D morphology.

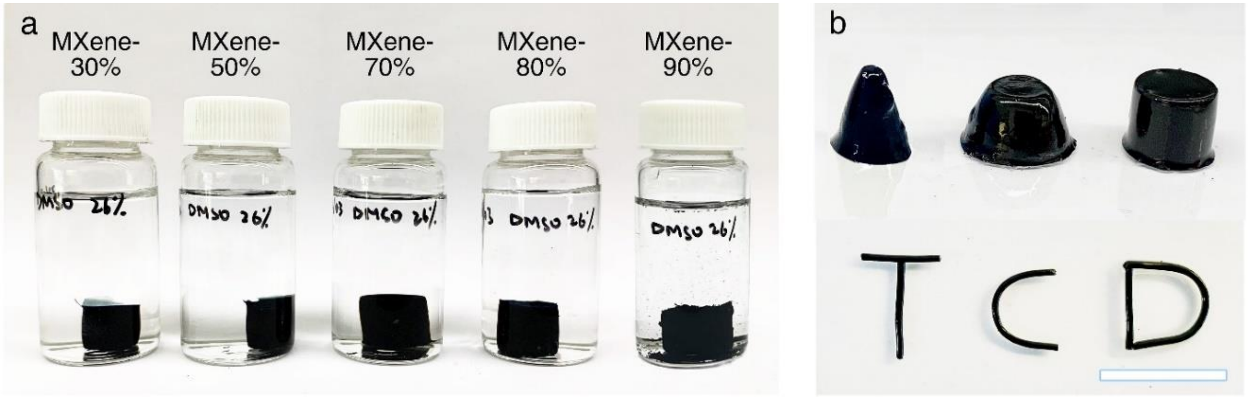

Supplementary Figure 3 | Photographs of MXene hydrogels. (a) Photographs of $Ti_3C_2T_x$ hydrogels with 30, 50, 70, 80, and 90 wt.% MXenes transferred from the original vials shown in Fig. 2a. (b) Photographs of $Ti_3C_2T_x$ MXene hydrogels (80 wt.%) with different shapes. The fiber-shaped hydrogels show great flexibility, which allows for patterning letters “TCD”. Scale bar: 1 cm.

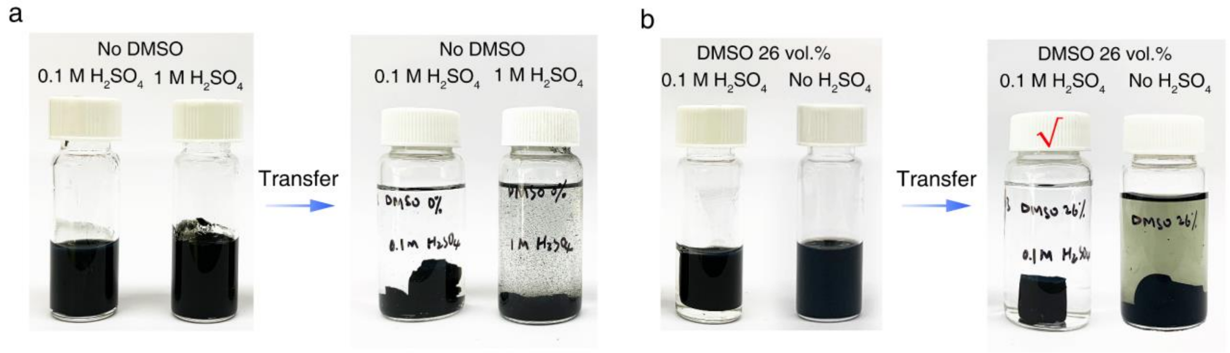

Supplementary Figure 4 | Photographs of MXene hydrogels. Photographs of $Ti_3C_2T_x$ MXene hydrogels self-assembled in different solutions. (a) Without DMSO, the as-prepared $Ti_3C_2T_x$ hydrogel in 0.1 M H_2SO_4 is weak, and hydrogel cannot even form in 1 M H_2SO_4. (b) Without H_2SO_4, the $Ti_3C_2T_x$ hydrogel in 26 vol.% DMSO is soft and easily broken, whereas in the mixture solution of 26 vol.% DMSO and 0.1 M H_2SO_4, the obtained $Ti_3C_2T_x$ hydrogel shows the best mechanical strength. The mass content of $Ti_3C_2T_x$ MXene in these experiments was 80 wt.%.

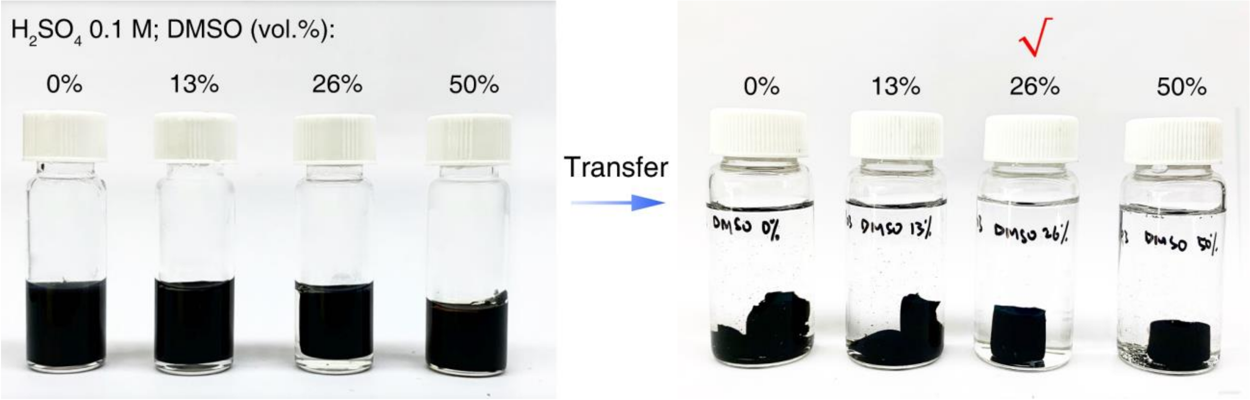

Supplementary Figure 5 | Photographs of MXene hydrogels. Photographs of $Ti_3C_2T_x$ hydrogels self-assembled in mixture solutions of 0.1 M H_2SO_4 and DMSO, the volumetric ratios of DMSO to the whole suspensions varied from 0% to 50%. In 26 vol.% DMSO, the self-assembled $Ti_3C_2T_x$ hydrogel shows the most evident swelling behavior and the best mechanical properties, no breakage occurred during the transferring process. The mass content of $Ti_3C_2T_x$ MXene in these experiments was 80 wt.%.

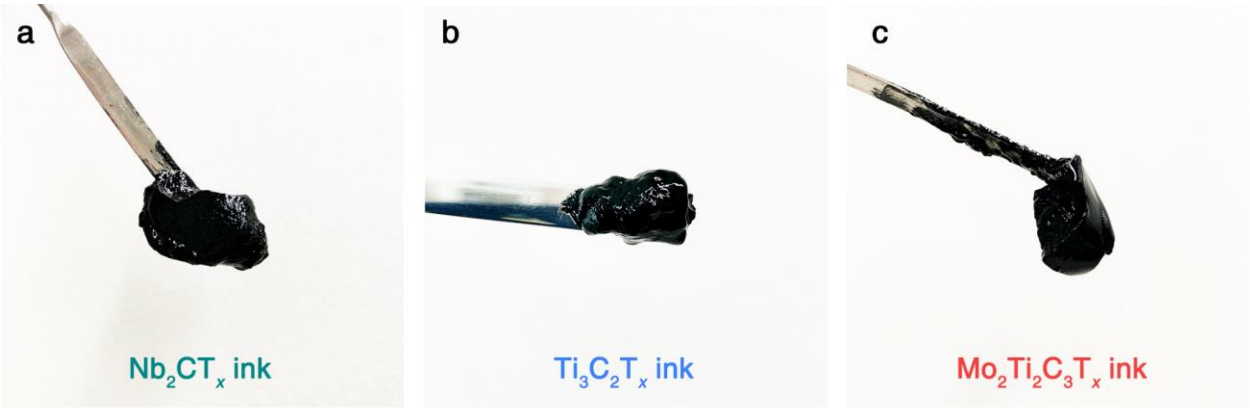

Supplementary Figure 6 | Photographs of solid-like MXene inks. (a) Nb_2CT_x ink, (b) $Ti_3C_2T_x$ ink, and (c) $Mo_2Ti_2C_3T_x$ ink. The concentration of MXene-PEDOT:PSS in three inks is ~50 mg mL^{-1}.

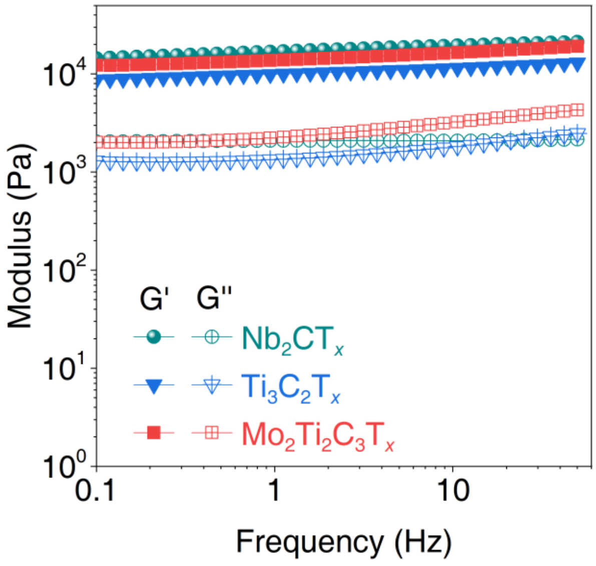

Supplementary Figure 7 | Rheological characterization of MXene inks. Storage modulus (G') and loss modulus (G'') of Nb_2CT_x, $Ti_3C_2T_x$, and $Mo_2Ti_2C_3T_x$ inks as functions of frequency.

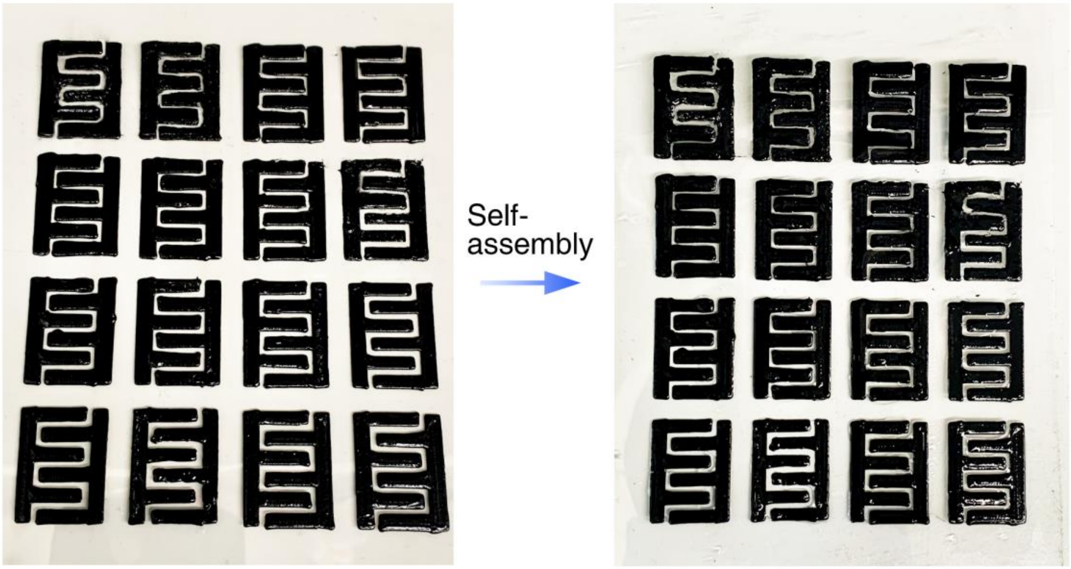

Supplementary Figure 8 | Photographs of MSC units. Scale-up 4D-printing of $Mo_2Ti_2C_3T_x$ hydrogel MSC units (size 2.2 cm × 1.7 cm × 0.05 cm (L × W × H)) on PET film. 16 MSC units were 3D printed in 0.5 h, which further transformed from sols into hydrogels after a self-assembly process. This process is consistently repeatable, more hydrogel units can be easily manufactured.

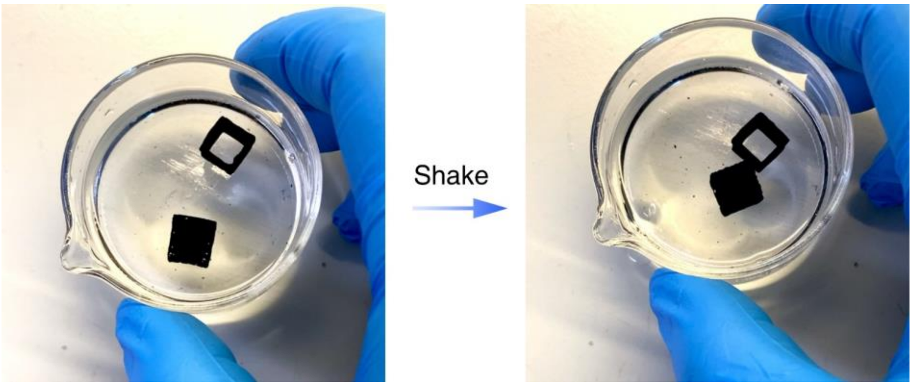

Supplementary Figure 9 | Photographs of MXene hydrogels. Photographs of 4D-printed $Ti_3C_2T_x$ hydrogel microlattice and rectangular hollow prism before and after shaking for ~14 s. After shaking, the two hydrogels retained their integrity.

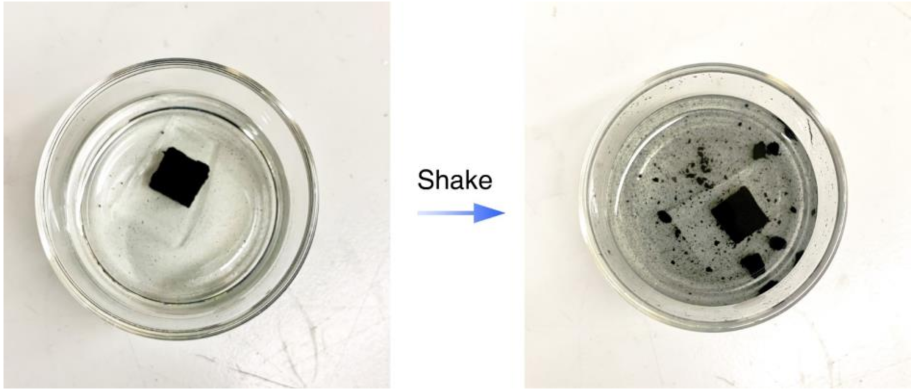

Supplementary Figure 10 | Photographs of MXene sol. Photographs of 3D-printed $Ti_3C_2T_x$ sol microlattice before and after shaking for ~8 s. After shaking, the sol microlattice broke into fragments. It is worth noting that, there are already some electrostatic attractions between the negatively charged MXenes and positively charged $PEDOT^+$ chains and protons, which protect this sol microlattice from complete redispersion in water. The pure $Ti_3C_2T_x$ sol architectures that only possess weak van der Waals interactions[4, 5] will be completely redispersed after shaking.

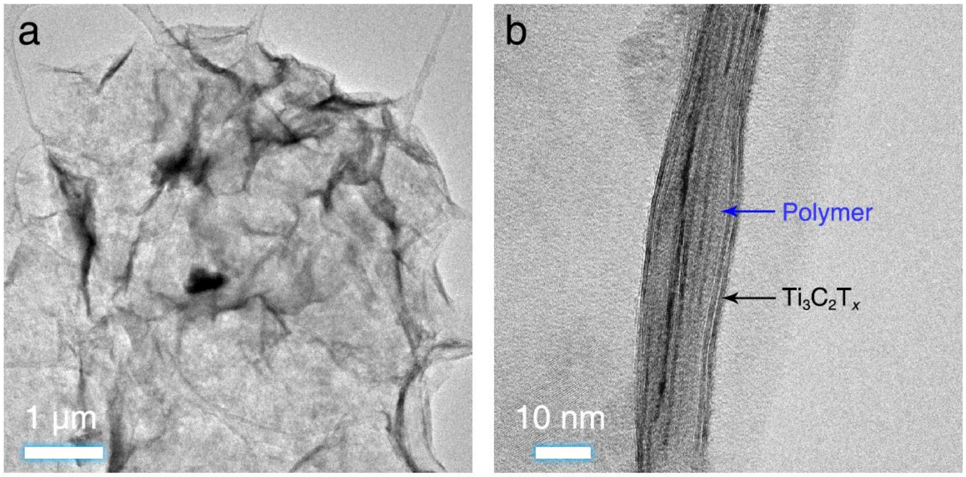

Supplementary Figure 11 | Morphology characterization of MXenes hydrogels. (a) Low- and (b) high-magnification TEM images of 4D-printed $Ti_3C_2T_x$ hydrogel. $Ti_3C_2T_x$ hydrogel shows a 3D structure with polymers sandwiched between $Ti_3C_2T_x$ layers.

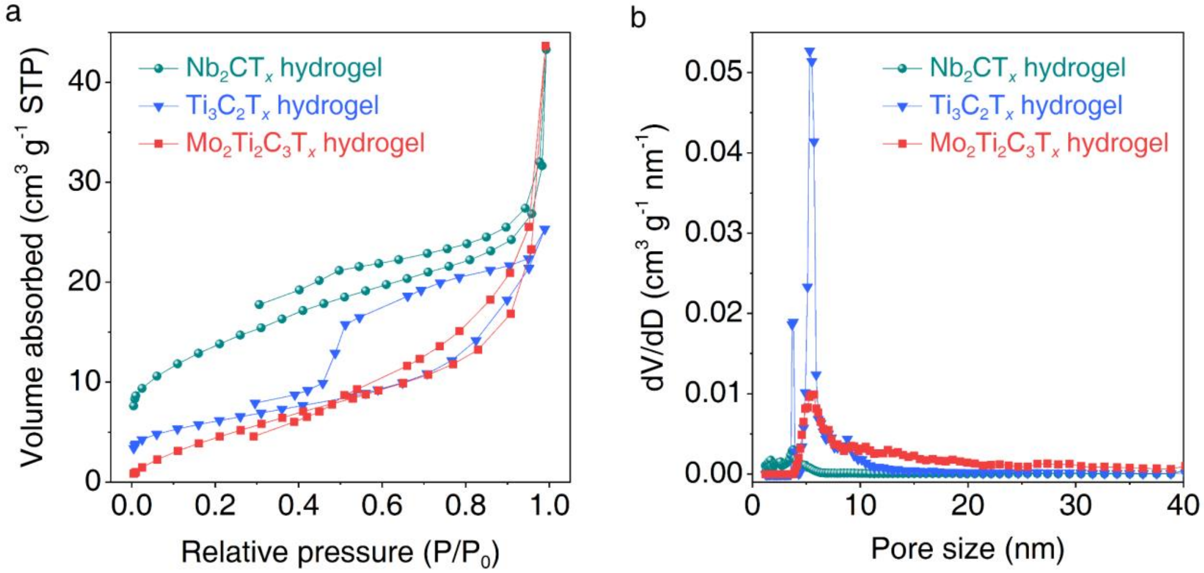

Supplementary Figure 12 | Specific surface area and pore size characterization of MXene hydrogels. (a) N_2 adsorption/desorption isotherms and (b) corresponding pore size distributions of 4D-printed Nb_2CT_x, $Ti_3C_2T_x$, and $Mo_2Ti_2C_3T_x$ hydrogels (vacuum-dried). The pore size distribution has been computed using DFT models in the Quantachrome ASiQwin software. The mesopore size of Nb_2CT_x hydrogel centers at ~4 nm, and the mesopore size of $Ti_3C_2T_x$ and $Mo_2Ti_2C_3T_x$ hydrogels centers at ~5.5 nm.

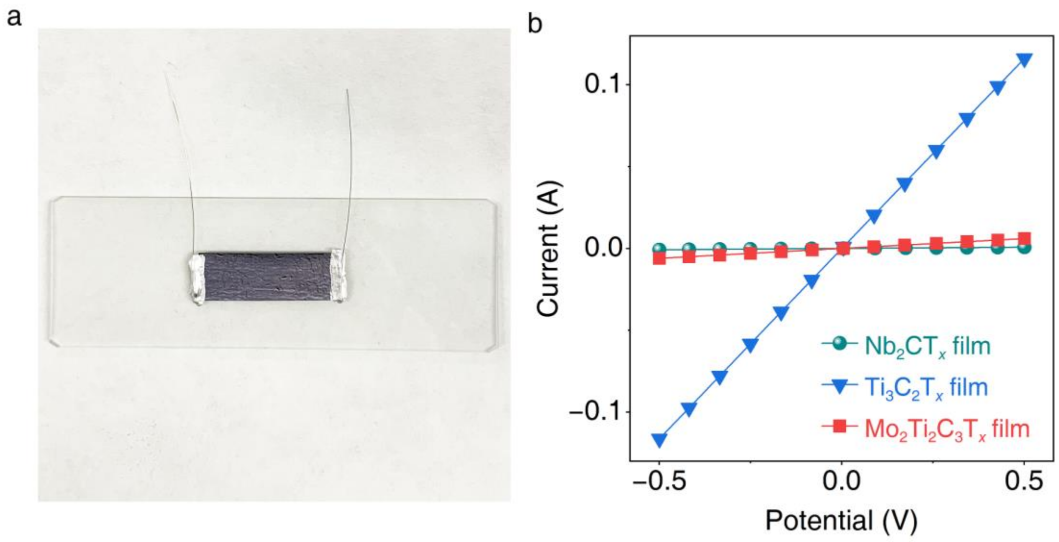

Supplementary Figure 13 | Electrical characterization of MXene films. (a) Photograph of electrical conductivity test for MXene films. Two silver wires were attached to MXene film using silver paste. (b) I-V curves of MXene films. The electrical conductivities of Nb_2CT_x film, $Ti_3C_2T_x$ film, and $Mo_2Ti_2C_3T_x$ film are 382 S m^{-1}, 58,149 S m^{-1}, and 3,018 S m^{-1}, respectively. These values are about an order of magnitude higher than their corresponding hydrogels, suggesting that the electrical conductivity of MXenes determines the electrical conductivity of MXene hydrogels.

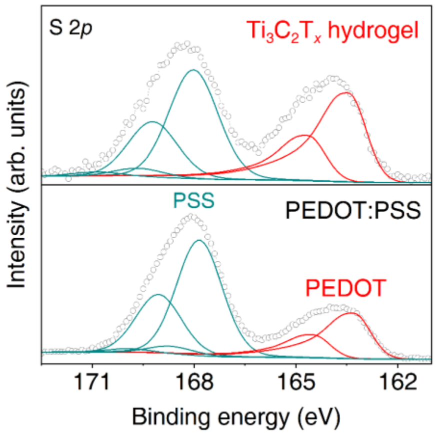

		PEDOT:PSS film		$Ti_3C_2T_x$ hydrogel	
Core level	Assignment	Position (eV)	Percentage	Position (eV)	Percentage
S $2p_{3/2}$	PSS	167.8	68.8%	168	53.2%
S $2p_{1/2}$	PSS	169		169.2	
S $2p_{3/2}$	PSS	168.7		168.7	
S $2p_{1/2}$	PSS	169.9		169.9	
S $2p_{3/2}$	PEDOT	163.3	31.2%	163.5	46.8%
S $2p_{1/2}$	PEDOT	164.5		164.7	

Supplementary Figure 14 | XPS analysis of MXene hydrogel and PEDOT:PSS film. High resolution S 2*p* XPS spectra of plain PEDOT:PSS film and $Ti_3C_2T_x$ hydrogel, and the peak assignments. Binding energies were all calibrated to the C 1*s* peak at 284.8 eV. The ratio of PSS to PEDOT reduces from 2.2 in PEDOT:PSS film to 1.1 in $Ti_3C_2T_x$ hydrogel. The assignments are referring to this reference[6].

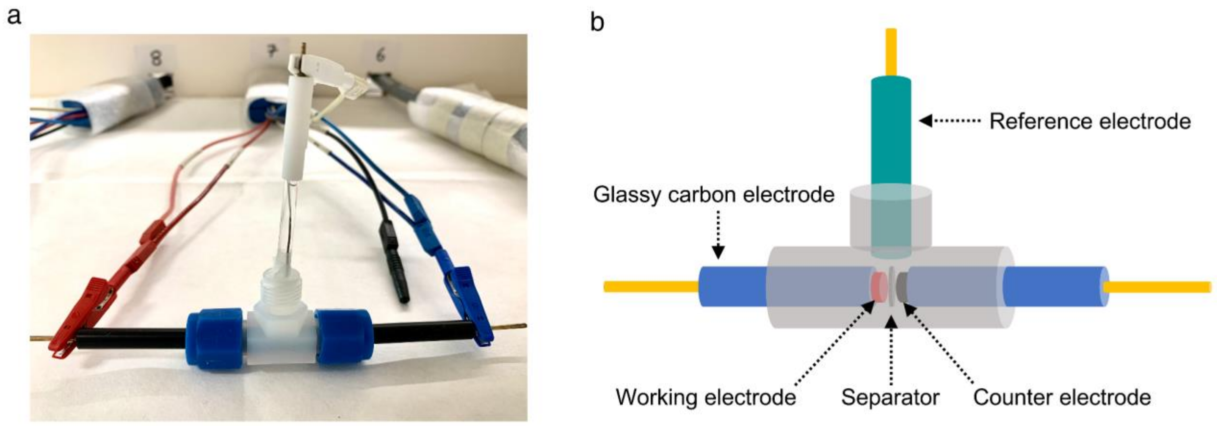

Supplementary Figure 15 | Swagelok cell. (a) Photograph and (b) schematic of Swagelok cell for three-electrode test.

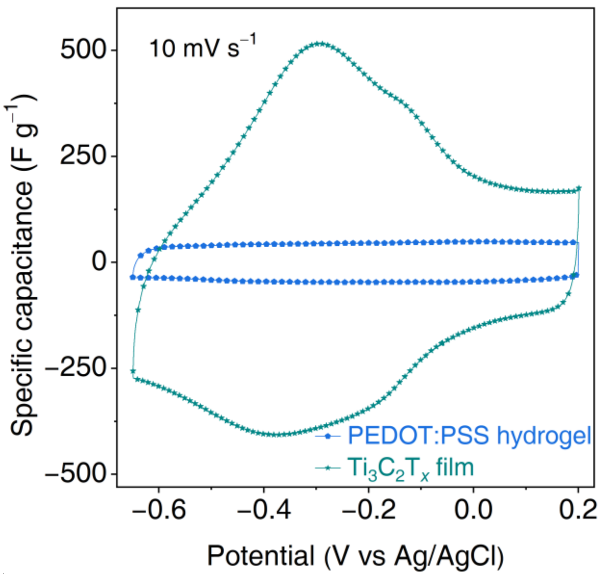

Supplementary Figure 16 | CV curves of PEDOT:PSS hydrogel and $Ti_3C_2T_x$ film. CV curves of PEDOT:PSS hydrogel and filtered $Ti_3C_2T_x$ film at a scan rate of 10 mV s^{-1}. Their mass loadings are ~1.5 mg cm^{-2}. The specific capacitances of $Ti_3C_2T_x$ film and PEDOT:PSS hydrogel are 281.8 F g^{-1} and 43.2 F g^{-1}, respectively.

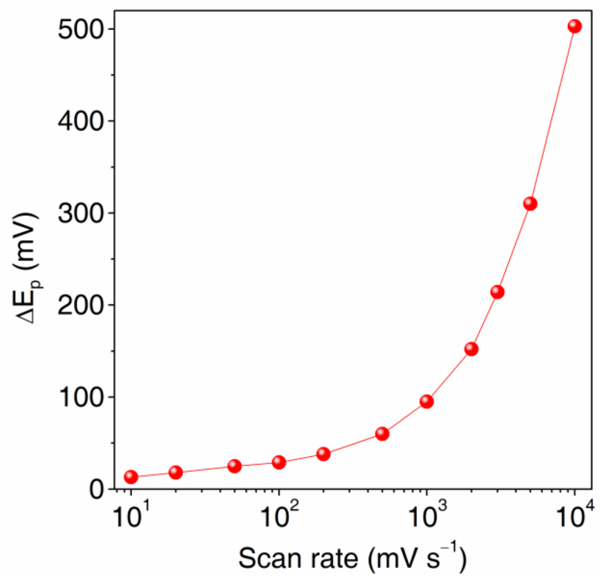

Supplementary Figure 17 | Peak potential separation of MXene hydrogel. Cathodic and anodic peak potential separation, ΔE_p, at different scan rates extracted from the CV curves of 4D-printed $Ti_3C_2T_x$ hydrogel (0.5 mg cm^{-2}).

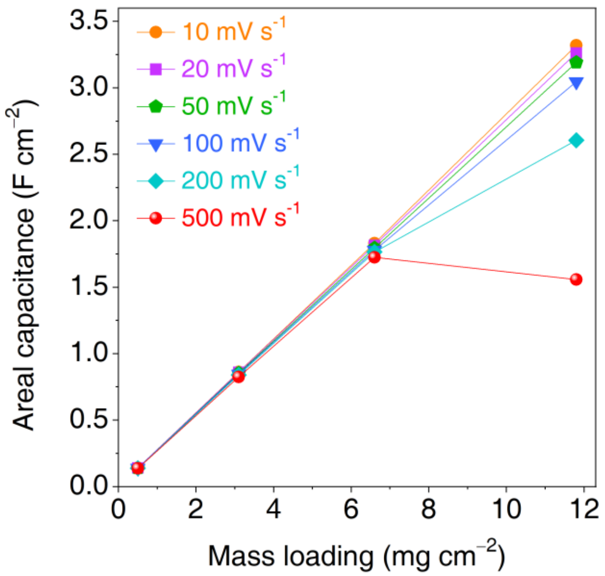

Supplementary Figure 18 | Areal capacitance of MXene hydrogels. Areal capacitances of 4D-printed $Ti_3C_2T_x$ hydrogels with different mass loadings at different scan rates.

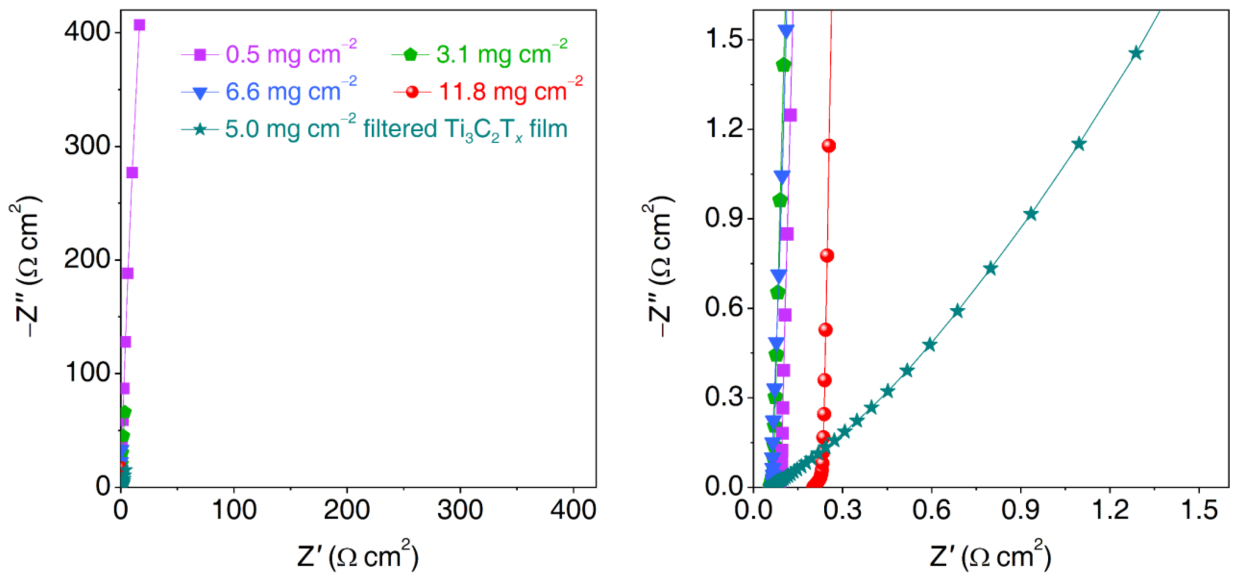

Supplementary Figure 19 | Electrochemical impedance spectra of MXene hydrogels and film. EIS plots of 4D-printed $Ti_3C_2T_x$ hydrogels with different mass loadings and a filtered $Ti_3C_2T_x$ film with a mass loading of 5.0 mg cm^{-1}.

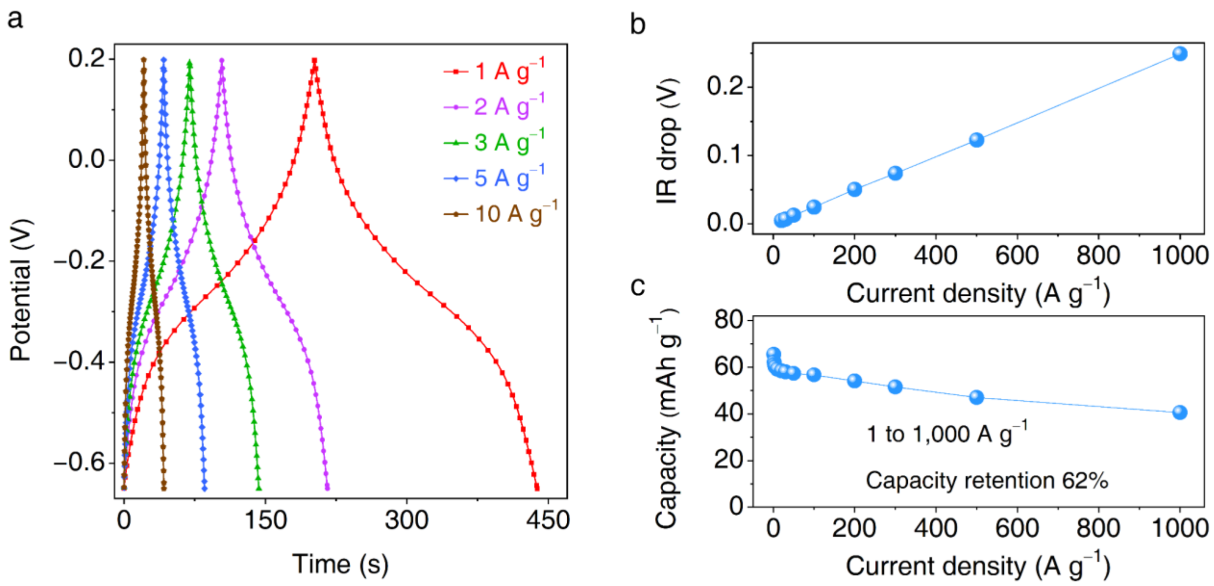

Supplementary Figure 20 | Electrochemical performance of MXene hydrogel. (a) GCD profiles of 4D-printed $Ti_3C_2T_x$ hydrogel (1.0 mg cm^{-2}) at current densities of 1, 2, 3, 5, and 10 A g^{-1}. (b) IR drop of 4D-printed $Ti_3C_2T_x$ hydrogel (1.0 mg cm^{-2}). (c) Rate performance of 4D-printed $Ti_3C_2T_x$ hydrogel (1.0 mg cm^{-2}).

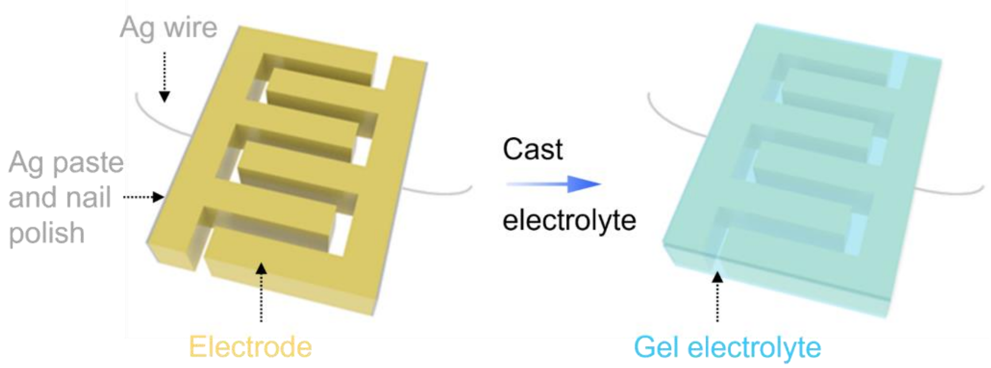

Supplementary Figure 21 | Schematic of MSC. Schematic of designing 4D-printed $Ti_3C_2T_x$ hydrogel MSC. The volume percentage of electrodes to the whole MSC is ~70 vol.%, and the gap between electrodes is ~30 vol.%. Because the electrodes are highly porous, they can absorb almost as much electrolyte as their volume. Thus, the volume of the gel electrolyte added should be at least the same as the volume of MSC (including both the electrodes and the gap). To ensure the complete infiltration of electrodes and maximize the electrochemical performance of MSC, the total volume of the cast PVA-EG-H_2SO_4 gel electrolyte was set to 120 vol.% of the MSC.

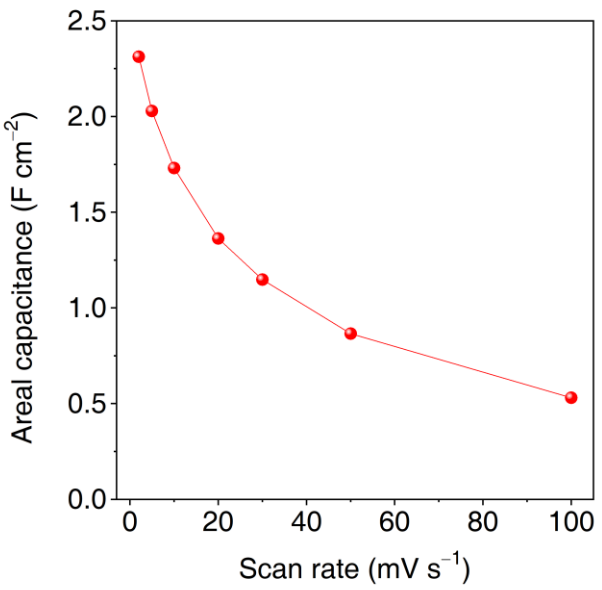

Supplementary Figure 22 | Rate capability of MSC. Rate performance of 4D-printed $Ti_3C_2T_x$ hydrogel MSC at scan rates from 2 to 100 mV s^{-1} at 25 °C.

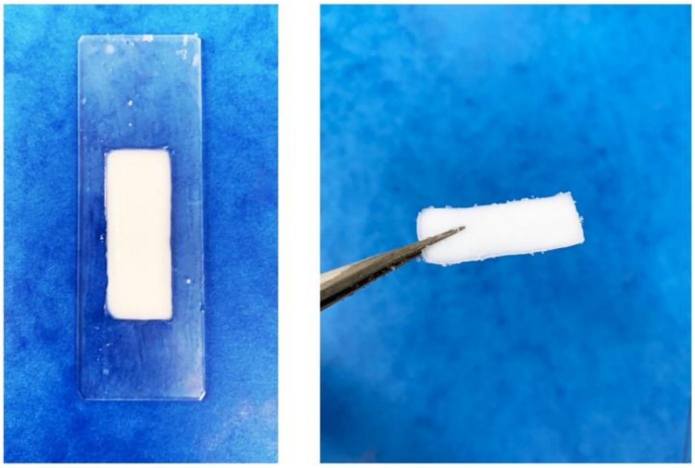

Supplementary Figure 23 | Photographs of PVA-H_2SO_4 electrolyte. Photographs of PVA-H_2SO_4 electrolyte at –20 °C. The white color suggests that the PVA-H_2SO_4 electrolyte is in a frozen state, which is more rigid than PVA-EG-H_2SO_4 gel electrolyte at the same temperature.

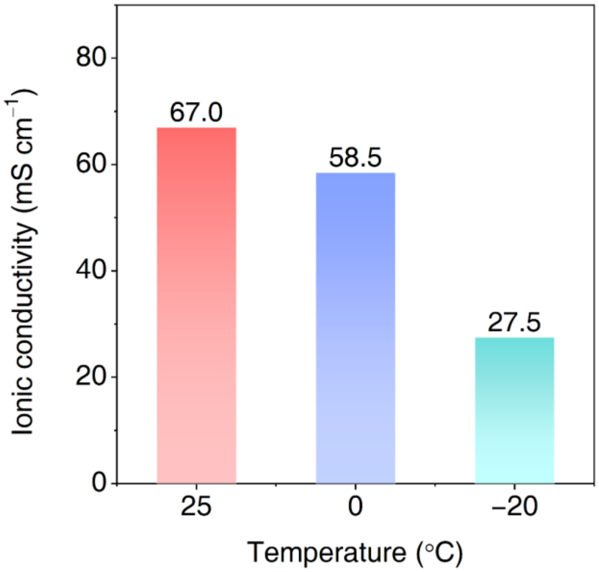

Supplementary Figure 24 | Ionic conductivity of PVA-EG-H_2SO_4 gel electrolyte. Ionic conductivity of PVA-EG-H_2SO_4 gel electrolyte at different temperatures.

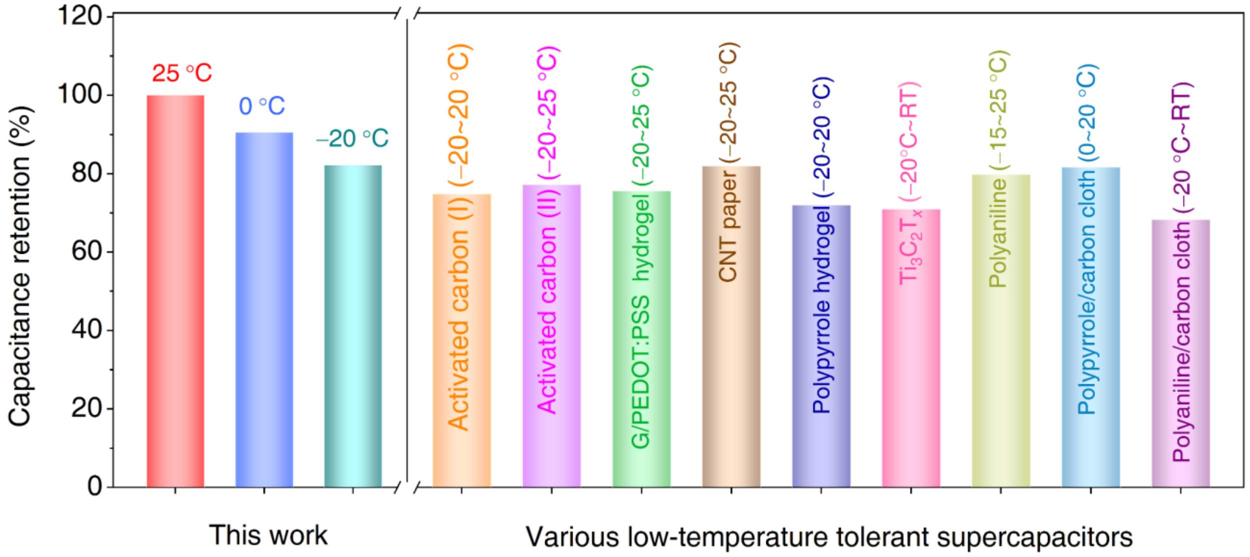

Supplementary Figure 25 | Capacitance retention of MSCs. Capacitance retention of 4D-printed $Ti_3C_2T_x$ hydrogel MSC in comparison with other low-temperature tolerant supercapacitors, including activated carbon SC (I)[7], activated carbon SC (II)[8], G/PEDOT:PSS hydrogel SC[9], CNT paper SC[10], polypyrrole hydrogel SC[11], $Ti_3C_2T_x$ SC[12], polyaniline SC[13], polypyrrole/carbon cloth SC[14], and polyaniline/carbon cloth SC[15]. RT: room temperature.

Supplementary Table 1. Comparison of 4D printing technology with traditional 3D printing on producing 3D MXene architectures

Printing technology	Ink formulation	Products	Properties	Reference
4D printing	MXenes (Nb_2CT_x, $Ti_3C_2T_x$, or $Mo_2Ti_2C_3T_x$) + PEDOT:PSS + DMSO + H_2SO_4 + Sodium L-ascorbate	Nb_2CT_x hydrogel	1. Hydrogel state.<br>2. Customizable geometries.<br>3. Strong interactions between MXenes and polymer chains.<br>4. Satisfying mechanical strength and operability.<br>5. Stable in water.<br>6. hydrogels can be used as electrodes directly.	This work
		$Ti_3C_2T_x$ hydrogel		
		$Mo_2Ti_2C_3T_x$ hydrogel		
3D printing	$Ti_3C_2T_x$	$Ti_3C_2T_x$ sol	1. Sol/ink state.<br>2. Customizable geometries.<br>3. Weak interactions between MXene flakes, or MXenes and other components.<br>4. Unstable in water (re-dispersible).<br>5. Freeze-drying is essential to maintain the shape of 3D printed patterns before conducting electrochemical tests.	4, 5, 16, 17
3D printing	$Ti_3C_2T_x$ + $ZnSO_4·7H_2O$	$Ti_3C_2T_x$ sol		18
3D printing	$Ti_3C_2T_x$ + cellulose nanofiber	$Ti_3C_2T_x$-cellulose nanofiber sol		19
3D printing	V_2CT_x + CNT + GO	V_2CT_x-CNT-GO sol		20
3D printing	NiCoP/$Ti_3C_2T_x$ + CNT	NiCoP/$Ti_3C_2T_x$-CNT sol		21

Supplementary Table 2. Comparison of electrical conductivity of various hydrogels and their application fields

Hydrogels	Electrical conductivity ($S\ m^{-1}$)	Applications	Reference
4D printed Nb_2CT_x hydrogel	37	NA	This work
4D printed $Ti_3C_2T_x$ hydrogel	1548	Supercapacitors	
4D printed $Mo_2Ti_2C_3T_x$ hydrogel	231	NA	
$Ti_3C_2T_x$ hydrogel	220	Solar steam generation	[22]
$Ti_3C_2T_x$/PVA hydrogel	~0.1	Capacitive deionization	[23]
$Ti_3C_2T_x$/PVA hydrogel	0.056	Sensors	[24]
$Ti_3C_2T_x$/poly(acrylamide-acrylic acid)/chitosan hydrogel	1.34	Sensors	[25]
$Ti_3C_2T_x$/cellulose nanofibrils/PAM hydrogel	0.19	Sensors	[26]
$Ti_3C_2T_x$/PAM/poly(N-isopropyl acrylamide) hydrogel	1.092	Sensors	[27]
$Ti_3C_2T_x$/PAM/PVA hydrogel	~0.06	Sensors	[28]
$Ti_3C_2T_x$/PAA hydrogel	3.8	Sensors	[29]
$Ti_3C_2T_x$/PAA hydrogel	0.8	Electromagnetic interference shielding	[30]
$Ti_3C_2T_x$/bacterial cellulose hydrogel	0.07	Wound healing	[31]
$Ti_3C_2T_x$/graphene/CNT hydrogel	175	Supercapacitors	[32]
PEDOT:PSS hydrogel	880	Supercapacitors	[33]
PEDOT:PSS hydrogel	10	Bioelectronics	[34]
PEDOT:PSS/PAA hydrogel	23	Bioelectronics	[35]
PEDOT:PSS/polypyrrole hydrogel	867	Biosensors	[36]
PEDOT:PSS/γ-polyglutamic acid hydrogel	12.5	Sensors	[37]

PEDOT:PSS/PAM/laponite hydrogel	26	Bioengineering	[38]
PEDOT:PSS/graphene oxide hydrogel	829.6	Bioelectronics	[39]
PEDOT:PSS/gold nanoparticle hydrogel	67000	Bioelectronics	[40]
PAA/polyaniline hydrogel	0.141	Bioengineering	[41]
P(urea-ionic liquid-3-sulfopropyl methacrylate potassium salt) hydrogel	3	Conductors	[42]
PVA/Aramid nanofibers/silver nanowire hydrogel	16600	Electromagnetic interference shielding	[43]
PAM/alginate/silver flake hydrogel	37400	Soft electronics	[44]
Graphene hydrogel	58	NA	[45]
Graphene hydrogel	0.5	Supercapacitors	[46]

Note: all the data are extracted from the reported values or figures in references.

PVA: polyvinyl alcohol; PAM: polyacrylamide; PAA: Polyacrylic acid.

Supplementary Table 3. Assignments of Ti 2*p* XPS spectra of $Ti_3C_2T_x$ film and $Ti_3C_2T_x$ hydrogel

		$Ti_3C_2T_x$ film		$Ti_3C_2T_x$ hydrogel	
Core level	Assignment	Position	Percentage	Position	Percentage
Ti $2p_{3/2}$	Ti (II)	455.5 eV	39%	456.4 eV	35.4%
Ti $2p_{1/2}$	Ti (II)	461.2 eV		462.1 eV	
Ti $2p_{3/2}$	Ti (III)	456.6 eV	26.5%	457.6 eV	20.6%
Ti $2p_{1/2}$	Ti (III)	462.3 eV		463.3 eV	
Ti $2p_{3/2}$	Ti−C	454.7 eV	25%	455.5 eV	33%
Ti $2p_{1/2}$	Ti−C	460.4 eV		461.2 eV	
Ti $2p_{3/2}$	Ti (IV)	457.9 eV	9.5%	459.2 eV	11%
Ti $2p_{1/2}$	Ti (IV)	463.6 eV		464.9 eV	

Note: Binding energies were all calibrated to the C 1*s* peak at 284.8 eV. The assignments are referring to these references[47, 48].

Supplementary Table 4. Assignments of C 1*s* XPS spectra of $Ti_3C_2T_x$ film and $Ti_3C_2T_x$ hydrogel

		$Ti_3C_2T_x$ film		$Ti_3C_2T_x$ hydrogel	
Core level	Assignment	Position	Percentage	Position	Percentage
C 1*s*	C−C	284.8 eV	55.9%	284.8 eV	49.9%
	C−Ti	281.8 eV	9.8%	282.3 eV	28.9%
	C−O	286.3 eV	28.5%	286.3 eV	18%
	C=O	287.8 eV	3.7%	287.8 eV	1.5%
	O−C=O	288.8 eV	2.1%	288.8 eV	1.7%

Note: Binding energies were all calibrated to the C 1*s* peak at 284.8 eV. The assignments are referring to these references[47, 48].

Supplementary Table 5. Comparison of electrochemical performance of various high-rate supercapacitor electrodes

Electrode	Mass loading (mg cm^{-2})	Areal capacitance at 10 mV s^{-1}	Capacitance retention from 10 to 1,000 mV s^{-1}	Specific capacitance at 10 V s^{-1}	Reference
4D printed $Ti_3C_2T_x$ hydrogel	0.5	0.14 F cm^{-2}	96.3%	232.9 F g^{-1}	This work
	3.1	0.86 F cm^{-2}	92.4%	104.4 F g^{-1}	
	6.6	1.83 F cm^{-2}	90.2%	51.8 F g^{-1}	
	11.8	3.32 F cm^{-2}	26.7%	NA	
Filtered porous $Ti_3C_2T_x$ film	0.53	0.1378 F cm^{-2}	87.3%	207.9 F g^{-1}	[49]
$Ti_3C_2T_x$/NbN film	1.05	0.29 F cm^{-2}	71%	78.8 F g^{-1}	[50]
$Ti_3C_2T_x$/rGO hydrogel	1.16	0.3423 F cm^{-2}	81%	NA	[51]
Filtered $Ti_3C_2T_x$ film	2.1	NA	43.6%	NA	[52]
Wavy $Ti_3C_2T_x$ film	2.28	0.7662 F cm^{-2}	88.9%	NA	
Oxidized $Ti_3C_2T_x$ film	1	0.34 F cm^{-2}	68.2%	71 F g^{-1}	[53]
	2.3	0.73 F cm^{-2}	41.5%	27 F g^{-1}	
	4.4	1.35 F cm^{-2}	28%	20 F g^{-1}	
	9.1	2.52 F cm^{-2}	22.6%	13 F g^{-1}	
	12	3.0 F cm^{-2}	22%	9.5 F g^{-1}	
Filtered $Ti_3C_2T_x$ hydrogel	1.2	0.42 F cm^{-2}	59%	37.8 F g^{-1}	[54]
	5.3	1.84 F cm^{-2}	13.5%	NA	
	11.3	3.7 F cm^{-2}	12.5%	NA	
Porous $Ti_3C_2T_x$	0.43	0.14 F cm^{-2}	92.8%	210 F g^{-1}	
	0.9	0.27 F cm^{-2}	87%	121.6 F g^{-1}	

	4.3	1.33 F cm^{-2}	41%	31 F g^{-1}	
Liquid-crystal $Ti_3C_2T_x$	2.8	0.76 F cm^{-2}	80.3%	126.2 F g^{-1}	[55]
	3.6	0.93 F cm^{-2}	65.4%	98.4 F g^{-1}	
	6.16	1.53 F cm^{-2}	42.8%	21.4 F g^{-1}	
1T-MoS_2 film	6.64	1.38 F cm^{-2}	53.6%	31 F g^{-1}	[56]
	10.87	2.01 F cm^{-2}	37.3%	12 F g^{-1}	
Graphene ribbon film	1	NA	53.2%	NA	[57]
$Ti_3C_2T_x$ hydrogel	NA	NA	87%	NA	[58]
$Ti_3C_2T_x$ hydrogel	NA	NA	50.3%	NA	[59]

Note: all the data are extracted from the reported values or figures in references.

Supplementary Table 6. Comparison of areal capacitance of various printed MSCs

Device	Electrolyte	Scan rate or current density	Areal capacitance (F cm^{-2})	Reference
4D-printed $Ti_3C_2T_x$ hydrogel MSC	PVA-EG-H_2SO_4	2 mV s^{-1}	2.31	This work
		5 mV s^{-1}	2.03	
		10 mV s^{-1}	1.73	
		20 mV s^{-1}	1.36	
		30 mV s^{-1}	1.15	
		50 mV s^{-1}	0.87	
		100 mV s^{-1}	0.53	
3D-printed $Ti_3C_2T_x$ MSC (I)	PVA-H_2SO_4	1.7 mA cm^{-2}	2.1	[4]
3D-printed G MSC	PVA-LiOH	2 mA cm^{-2}	1.57	[60]
3D-printed PANI/rGO MSC	PVA-H_2SO_4	3.4 mA cm^{-2}	1.329	[61]
3D-printed $Ti_3C_2T_x$ MSC (II)	PVA-H_2SO_4	2 mV s^{-1}	1.035	[5]
3D-printed $Ti_3C_2T_x$/Ag NW/MnONW/C60 MSC	PVA-KOH	10 mV s^{-1}	0.2478	[62]
3D-printed VO_x/rGO// G-VNQDs/rGO MSC	PVA-LiCl	0.63 mA cm^{-2}	0.2079	[63]
Screen-printed $Ti_3C_2T_x$ MSC	PVA-H_2SO_4	0.08 mA cm^{-2}	0.158	[64]
Screen-printed PEDOT:PSS/MnO_2 MSC	PVA-LiCl	0.08 mA cm^{-2}	0.1354	[65]
3D-printed CNT MSC	PVA-H_3PO_4	50 mV s^{-1}	0.00469	[66]
Inkjet-printed $Ti_3C_2T_x$ MSC	PVA-H_2SO_4	1 A g^{-1}	0.1081	[67]
Inkjet-printed Ni@MnO_2 MSC	PVA-CH_3COOLi	5 mV s^{-1}	0.0529	[68]
Inkjet-printed N-doped carbon MSC	PVA-H_2SO_4	5 mV s^{-1}	0.0039	[69]

Note: all the data are extracted from the reported values or figures in references.

G: graphene; PANI: polyaniline; rGO: reduced graphene oxide; AgNW: Ag nanowire; MnONW: MnO_2 nanowire; C60: fullerene; VNOQs: vanadium nitride quantum dots; CNT: carbon nanotube.

Supplementary Table 7. Comparison of areal energy and power densities of various MSCs

Device	Voltage window (V)	Areal power density (mW cm^{-2})	Areal energy density (μWh cm^{-2})	Reference
4D-printed $Ti_3C_2T_x$ hydrogel MSC	0.6	0.21	92.88	This work
		0.42	90.83	
		0.63	88.37	
		1.04	83.28	
		2.014	71.58	
		3.77	53.07	
		5.21	39.01	
		6.96	20.16	
3D-printed $Ti_3C_2T_x$ MSC (I)	0.6	0.26	26	4
		0.64	24.4	
		1.35	23	
		2.6	19.5	
		3.85	17	
		6.5	12	
3D-printed $Ti_3C_2T_x$ MSC (II)	0.6	0.62	51.7	5
		1.4	45	
		2.1	35.8	
		2.7	25	
		3.6	12.7	
		4	6.85	
Screen-printed $Ti_3C_2T_x$ MSC	0.6	0.01846	1.64	64
		0.03711	1.62	
		0.07557	1.57	
		0.15698	1.51	
		0.33499	1.42	
		0.54412	1.36	

		0.77833	1.32	
$RuO_2//Ti_3C_2T_x$ (In plane) MSC	1.5	0.3	30	70
		0.58	28.5	
		1.05	26	
		1.5	19	
Extrusion-printed $Ti_3C_2T_x$ MSC	0.5	0.0114	0.3166	71
		0.0225	0.312	
		0.0441	0.3065	
		0.0751	0.2086	
		0.1154	0.1603	
		0.1577	0.1094	
3D-printed VO_x/rGO// G-VNQDs/rGO MSC	1.6	0.5	73.9	63
		3.77	45	
Direct-written G-CNT MSC	1	0.026	1.36	72
		0.053	1.26	
		0.117	1.18	
		0.182	1.14	
		0.25	1.12	
MoS_2@rGO-CNT MSC	1	1.05	3.7	73
screen-printed G MSC	1.8	1.13	0.361	74
CNF@MnO_2//CNF@Fe_2O_3 MSC	2	0.55	26	75
		1.05	20	
		1.8	10.5	
		2.2	4	
		2.9	0.7	
Laser-patterned AgNWs-MoS_2 MSC	1.2	1.472	2.453	76

Note: all the data are extracted from the reported values or figures in references.

Supplementary Table 8. Comparison of ionic conductivity of various low-temperature tolerant gel electrolytes

Electrolyte	Temperature (°C)	Ionic conductivity (mS cm^{-1})	Reference
PVA-EG-H_2SO_4	25	67	This work
	0	58.5	
	–20	27.5	
PAM-EG-LiCl	20	~13.6	10
	0	~11.6	
	–20	~6.2	
	–40	2.38	
PVA-EG-glycerol-H_2SO_4	20	~127	9
	0	~81	
	–20	~53	
	–30	16	
PVA-EG-Zn(Tf)$_2$	RT	15.03	77
	–20	9.05	
	–40	3.53	
PVA-H_3PO_4	0	1.25	14
PVA-KOH	25	97	78
	0	89	
	–20	60	
PAM-EG-H_2SO_4	–30	13	13
PAM-PVP-H_3PO_4	30	97	79
	0	~70	
	–40	49	
P(AM-co-DMAEMA)-AMP-gelatin-LiCl	25	13.6	8
	0	~8.1	
	–20	~7.4	
	–40	4.3	

PAMAA-chitosan-NaCl-$LiSO_4$	RT	48	12
	–20	36	
PVA-PAMAA-glycerol-NaCl	–20	13.14	80
PAMPS-PAM-DMSO-LiCl	–20	8.2	81
PIP13FSI- PYR14FSI-SiO_2	20	5.5	7
	0	~2	
	–20	~0.8	
Poly(vinylidenefluoride-co-hexafluoropropylene)-EMITf-$Al(Tf)_3$	RT	~1.6	82
	–20	~0.8	

Note: all the data are extracted from the reported values or figures in references.

PAM: polyacrylamide; PAA: Polyacrylic acid; PVP: Polyvinylpyrrolidone; P(AM-co-DMAEMA): poly(acrylamide-co-2-(dimethylamino)ethylmethacrylate); AMP: adenosine monophosphate; PAMAA: poly(acrylic amide-acrylic acid); PAMPS: poly(2-acrylamido-2-methypropane sulfonic acid)

Supplementary Table 9. Comparison of areal capacitance of various supercapacitors at different temperatures

Device	Electrolyte	Temperature (°C)	Areal capacitance ($F\ cm^{-2}$)		Reference
4D-printed $Ti_3C_2T_x$ hydrogel MSC	PVA-EG-H_2SO_4	25	1.73	10 mVs^{-1}	This work
		0	1.57		
		–20	1.42		
Activated carbon SC (I)	PIP13FSI-PYR14FSI-SiO_2	20	0.238	5 mVs^{-1}	7
		0	0.233		
		–20	0.178		
Activated carbon SC (II)	P(AM-co-DMAEMA)-AMP-gelatin-LiCl	25	0.1636	1 mA cm^{-2}	8
		–20	0.1263		
G/PEDOT:PSS hydrogel	PVA-EG-glycerol-H_2SO_4	25	0.2628	0.1 A g^{-1}	9
		0	0.23337		
		–20	0.19869		
CNT paper SC	PAM-EG-LiCl	25	0.0167	0.1 A g^{-1}	10
		–20	0.0137		
Polypyrrole hydrogel SC	PVA-H_2SO_4	20	0.95	1.6 mA cm^{-2}	11
		0	0.893		
		–20	0.684		
$Ti_3C_2T_x$ SC	PAMAA-chitosan-NaCl-$LiSO_4$	RT	0.21	1 mA cm^{-2}	12
		–20	0.15		
PANI SC	PAM-EG-H_2SO_4	20	0.0144	0.03 mA cm^{-2}	13
		0	0.0127		
		–15	0.0115		
Polypyrrole/ carbon cloth SC	PVA-H_3PO_4	20	0.0648	0.5 mA cm^{-2}	14
		0	0.05291		

CNT/PANI SC	PAMPS-PAM-DMSO-LiCl	25	0.26	3.33 mA cm^{-2}	[81]
		0	0.2425		
		–20	0.2366		

Note: all the data are extracted from the reported values or figures in references.

Supplementary references

1. Wang Y, *et al.* Niobium carbide MXenes with broad-band nonlinear optical response and ultrafast carrier dynamics. *ACS Nano* **14**, 10492-10502 (2020).
2. Shahzad F, *et al.* Electromagnetic interference shielding with 2D transition metal carbides (MXenes). *Science* **353**, 1137-1140 (2016).
3. Anasori B, *et al.* Control of Electronic Properties of 2D Carbides (MXenes) by Manipulating Their Transition Metal Layers. *Nanoscale Horiz.* **1**, 227-234 (2016).
4. Yang W, *et al.* 3D Printing of Freestanding MXene Architectures for Current-Collector-Free Supercapacitors. *Adv. Mater.* **31**, 1902725 (2019).
5. Orangi J, Hamade F, Davis VA, Beidaghi M. 3D Printing of Additive-Free 2D $Ti_3C_2T_x$ (MXene) Ink for Fabrication of Micro-Supercapacitors with Ultra-High Energy Densities. *ACS Nano* **14**, 640-650 (2020).
6. Solazzo M, Krukiewicz K, Zhussupbekova A, Fleischer K, Biggs MJ, Monaghan MG. PEDOT:PSS interfaces stabilised using a PEGylated crosslinker yield improved conductivity and biocompatibility. *Journal of Materials Chemistry B* **7**, 4811-4820 (2019).
7. Nègre Lo, Daffos B, Turq V, Taberna P-L, Simon P. Ionogel-based solid-state supercapacitor operating over a wide range of temperature. *Electrochim. Acta* **206**, 490-495 (2016).
8. Zhang Q, *et al.* Nucleotide-Tackified Organohydrogel Electrolyte for Environmentally Self-Adaptive Flexible Supercapacitor with Robust Electrolyte/Electrode Interface. *Small* **17**, 2103091 (2021).
9. Xu T, Yang D, Zhang S, Zhao T, Zhang M, Yu Z-Z. Antifreezing and stretchable all-gel-state supercapacitor with enhanced capacitances established by graphene/PEDOT-polyvinyl alcohol hydrogel fibers with dual networks. *Carbon* **171**, 201-210 (2021).
10. Rong Q, Lei W, Huang J, Liu M. Low temperature tolerant organohydrogel electrolytes for flexible solid-state supercapacitors. *Adv. Energy Mater.* **8**, 1801967 (2018).
11. Chen F, Chen Q, Song Q, Lu H, Ma M. Strong and stretchable polypyrrole hydrogels with biphase microstructure as electrodes for substrate-free stretchable supercapacitors. *Adv. Mater. Interfaces* **6**, 1900133 (2019).
12. Peng J, *et al.* A mechanically robust all-solid-state supercapacitor based on a highly conductive double-network hydrogel electrolyte and $Ti_3C_2T_x$ MXene electrode with anti-freezing property. *J. Mater. Chem. A* **9**, 25073-25085 (2021).
13. Jin X, *et al.* Stretchable supercapacitor at −30 °C. *Energy Environ. Sci.* **14**, 3075-3085 (2021).
14. Liu J-h, Xu X-y, Liu C, Chen D-Z. Thermal effect on the pseudocapacitive behavior of high-performance flexible supercapacitors based on polypyrrole-decorated carbon cloth electrodes. *New J. Chem.* **45**, 12435-12447 (2021).
15. Qin G, *et al.* Multifunctional supramolecular gel polymer electrolyte for self-healable and cold-resistant supercapacitor. *J. Power Sources* **474**, 228602 (2020).
16. Tetik H, *et al.* 3D Printed MXene Aerogels with Truly 3D Macrostructure and Highly Engineered Microstructure for Enhanced Electrical and Electrochemical Performance. *Adv. Mater.* **34**, 2104980 (2022).
17. Shen K, Li B, Yang S. 3D printing dendrite-free lithium anodes based on the nucleated MXene arrays. *Energy Storage Mater.* **24**, 670-675 (2020).

18. Fan Z, *et al.* 3D-printed Zn-ion hybrid capacitor enabled by universal divalent cation-gelated additive-free Ti_3C_2 MXene ink. *ACS Nano* **15**, 3098-3107 (2021).
19. Zhou G, Li MC, Liu C, Wu Q, Mei C. 3D Printed $Ti_3C_2T_x$ MXene/Cellulose Nanofiber Architectures for Solid‐State Supercapacitors: Ink Rheology, 3D Printability, and Electrochemical Performance. *Adv. Funct. Mater.* **32**, 2109593 (2022).
20. Wang Z, *et al.* 3D-Printed Sodiophilic V_2CT_x/rGO-CNT MXene Microgrid Aerogel for Stable Na Metal Anode with High Areal Capacity. *ACS Nano* **16**, 9105-9116 (2022).
21. Yu L, Li W, Wei C, Yang Q, Shao Y, Sun J. 3D printing of NiCoP/Ti_3C_2 MXene architectures for energy storage devices with high areal and volumetric energy density. *Nano-Micro Lett.* **12**, 143 (2020).
22. Chen H, Ma H, Zhang P, Wen Y, Qu L, Li C. Pristine titanium carbide MXene hydrogel matrix. *ACS Nano* **14**, 10471-10479 (2020).
23. Ai J, Li J, Li K, Yu F, Ma J. Highly flexible, self-healable and conductive poly (vinyl alcohol)/$Ti_3C_2T_x$ MXene film and it's application in capacitive deionization. *Chem. Eng. J.* **408**, 127256 (2021).
24. Feng Y, *et al.* Muscle‐Inspired MXene Conductive Hydrogels with Anisotropy and Low‐Temperature Tolerance for Wearable Flexible Sensors and Arrays. *Adv. Funct. Mater.* **31**, 2105264 (2021).
25. Li S-N, *et al.* Environmentally stable, mechanically flexible, self-adhesive, and electrically conductive $Ti_3C_2T_x$ MXene hydrogels for wide-temperature strain sensing. *Nano Energy* **90**, 106502 (2021).
26. Wei Y, *et al.* MXene‐based conductive organohydrogels with long‐term environmental stability and multifunctionality. *Adv. Funct. Mater.* **30**, 2005135 (2020).
27. Zhang Y, *et al.* High-Strength, Self-Healable, Temperature-Sensitive, MXene-Containing Composite Hydrogel as a Smart Compression Sensor. *ACS Appl. Mater. Interfaces* **11**, 47350-47357 (2019).
28. Liao H, Guo X, Wan P, Yu G. Conductive MXene nanocomposite organohydrogel for flexible, healable, low‐temperature tolerant strain sensors. *Adv. Funct. Mater.* **29**, 1904507 (2019).
29. Li Y, Yan J, Liu Y, Xie X-M. Super Tough and Intelligent Multibond Network Physical Hydrogels Facilitated by $Ti_3C_2T_x$ MXene Nanosheets. *ACS Nano* **16**, 1567-1577 (2021).
30. Zhu Y, Liu J, Guo T, Wang JJ, Tang X, Nicolosi V. Multifunctional $Ti_3C_2T_x$ MXene composite hydrogels with strain sensitivity toward absorption-dominated electromagnetic-interference shielding. *ACS Nano* **15**, 1465-1474 (2021).
31. Mao L, *et al.* Biodegradable and electroactive regenerated bacterial cellulose/MXene ($Ti_3C_2T_x$) composite hydrogel as wound dressing for accelerating skin wound healing under electrical stimulation. *Adv. Healthc. Mater.* **9**, 2000872 (2020).
32. Yang X, *et al.* 3D Macroporous Oxidation‐Resistant $Ti_3C_2T_x$ MXene Hybrid Hydrogels for Enhanced Supercapacitive Performances with Ultralong Cycle Life. *Adv. Funct. Mater.* **32**, 2109479 (2022).
33. Yao B, *et al.* Ultrahigh-Conductivity Polymer Hydrogels with Arbitrary Structures. *Adv. Mater.* **29**, 1700974 (2017).
34. Zhang SM, *et al.* Room-Temperature-Formed PEDOT:PSS Hydrogels Enable Injectable, Soft, and Healable Organic Bioelectronics. *Adv. Mater.* **32**, 1904752 (2020).

35. Feig VR, Tran H, Lee M, Bao ZA. Mechanically tunable conductive interpenetrating network hydrogels that mimic the elastic moduli of biological tissue. *Nat. Commun.* **9**, 2740 (2018).
36. Ren X, *et al.* Highly conductive PPy–PEDOT: PSS hybrid hydrogel with superior biocompatibility for bioelectronics application. *ACS Appl. Mater. Interfaces* **13**, 25374-25382 (2021).
37. Zhang C, *et al.* Highly adhesive and self-healing γ-PGA/PEDOT:PSS conductive hydrogels enabled by multiple hydrogen bonding for wearable electronics. *Nano Energy* **95**, 106991 (2022).
38. Tondera C, *et al.* Highly Conductive, Stretchable, and Cell-Adhesive Hydrogel by Nanoclay Doping. *Small* **15**, 1901406 (2019).
39. Gan D, *et al.* Graphene Oxide-Templated Conductive and Redox-Active Nanosheets Incorporated Hydrogels for Adhesive Bioelectronics. *Adv. Funct. Mater.* **30**, 1907678 (2020).
40. Won D, *et al.* Digital selective transformation and patterning of highly conductive hydrogel bioelectronics by laser-induced phase separation. *Sci. Adv.* **8**, eabo3209 (2022).
41. Hosseinzadeh S, Rezayat SM, Vashegani-Farahani E, Mahmoudifard M, Zamanlui S, Soleimani M. Nanofibrous hydrogel with stable electrical conductivity for biological applications. *Polymer* **97**, 205-216 (2016).
42. Long T, Li Y, Fang X, Sun J. Salt-Mediated Polyampholyte Hydrogels with High Mechanical Strength, Excellent Self-Healing Property, and Satisfactory Electrical Conductivity. *Adv. Funct. Mater.* **28**, 1804416 (2018).
43. Zhou Q, *et al.* Mechanically strong and multifunctional hybrid hydrogels with ultrahigh electrical conductivity. *Adv. Funct. Mater.* **31**, 2104536 (2021).
44. Ohm Y, Pan C, Ford MJ, Huang X, Liao J, Majidi C. An electrically conductive silver–polyacrylamide–alginate hydrogel composite for soft electronics. *Nature Electronics* **4**, 185-192 (2021).
45. Yang X, Qiu L, Cheng C, Wu Y, Ma ZF, Li D. Ordered gelation of chemically converted graphene for next-generation electroconductive hydrogel films. *Angew. Chem. Int. Ed.* **50**, 7325-7328 (2011).
46. Xu Y, Sheng K, Li C, Shi G. Self-assembled graphene hydrogel via a one-step hydrothermal process. *ACS Nano* **4**, 4324-4330 (2010).
47. Shah SA, *et al.* Template-free 3D titanium carbide ($Ti_3C_2T_x$) MXene particles crumpled by capillary forces. *Chem. Commun.* **53**, 400-403 (2017).
48. Halim J, *et al.* X-ray photoelectron spectroscopy of select multi-layered transition metal carbides (MXenes). *Appl. Surf. Sci.* **362**, 406-417 (2016).
49. Kong J, *et al.* High-Mass-Loading Porous $Ti_3C_2T_x$ Films for Ultrahigh-Rate Pseudocapacitors. *ACS Energy Lett.* **5**, 2266-2274 (2020).
50. Wang H, *et al.* Enhanced Rate Capability of Ion-Accessible $Ti_3C_2T_x$-NbN Hybrid Electrodes. *Adv. Energy Mater.* **10**, 2001411 (2020).
51. Wu Z, *et al.* Reassembly of MXene hydrogels into flexible films towards compact and ultrafast supercapacitors. *Adv. Funct. Mater.* **31**, 2102874 (2021).
52. Li K, *et al.* All-pseudocapacitive asymmetric MXene-carbon-conducting polymer supercapacitors. *Nano Energy* **75**, 104971 (2020).

53. Tang J, *et al.* Optimizing Ion Pathway in Titanium Carbide MXene for Practical High-Rate Supercapacitor. *Adv. Energy Mater.* **11**, 2003025 (2021).
54. Lukatskaya MR, *et al.* Ultra-high-rate pseudocapacitive energy storage in two-dimensional transition metal carbides. *Nat. Energy* **2**, 17105 (2017).
55. Xia Y, *et al.* Thickness-independent capacitance of vertically aligned liquid-crystalline MXenes. *Nature* **557**, 409-412 (2018).
56. Chen W, *et al.* Two-dimensional quantum-sheet films with sub-1.2 nm channels for ultrahigh-rate electrochemical capacitance. *Nat. Nanotech.* **17**, 153-158 (2022).
57. Sheng L, *et al.* Multilayer‐Folded Graphene Ribbon Film with Ultrahigh Areal Capacitance and High Rate Performance for Compressible Supercapacitors. *Adv. Funct. Mater.* **28**, 1800597 (2018).
58. Deng Y, *et al.* Fast Gelation of $Ti_3C_2T_x$ MXene Initiated by Metal Ions. *Adv. Mater.* **31**, 1902432 (2019).
59. Huang X, Huang J, Yang D, Wu P. A Multi‐Scale Structural Engineering Strategy for High‐Performance MXene Hydrogel Supercapacitor Electrode. *Adv. Sci.* **8**, 2101664 (2021).
60. Tagliaferri S, *et al.* Aqueous Inks of Pristine Graphene for 3D Printed Microsupercapacitors with High Capacitance. *ACS Nano* **15**, 15342-15353 (2021).
61. Wang ZS, *et al.* Three-Dimensional Printing of Polyaniline/Reduced Graphene Oxide Composite for High-Performance Planar Supercapacitor. *ACS Appl. Mater. Interfaces* **10**, 10437-10444 (2018).
62. Li XR, Li HP, Fan XQ, Shi XL, Liang JJ. 3D-Printed Stretchable Micro-Supercapacitor with Remarkable Areal Performance. *Adv. Energy Mater.* **10**, 1903794 (2020).
63. Shen K, Ding JW, Yang SB. 3D Printing Quasi-Solid-State Asymmetric Micro-Supercapacitors with Ultrahigh Areal Energy Density. *Adv. Energy Mater.* **8**, 1800408 (2018).
64. Abdolhosseinzadeh S, Schneider R, Verma A, Heier J, Nuesch F, Zhang CF. Turning Trash into Treasure: Additive Free MXene Sediment Inks for Screen-Printed Micro-Supercapacitors. *Adv. Mater.* **32**, 2000716 (2020).
65. Li D, Yang S, Chen X, Lai WY, Huang W. 3D Wearable Fabric‐Based Micro‐Supercapacitors with Ultra‐High Areal Capacitance. *Adv. Funct. Mater.* **31**, 2107484 (2021).
66. Yu W, Zhou H, Li BQ, Ding S. 3D printing of carbon nanotubes-based microsupercapacitors. *ACS Appl. Mater. Interfaces* **9**, 4597-4604 (2017).
67. Wu CW, Unnikrishnan B, Chen IWP, Harroun SG, Chang HT, Huang CC. Excellent oxidation resistive MXene aqueous ink for micro-supercapacitor application. *Energy Storage Mater.* **25**, 563-571 (2020).
68. Lin Y, Gao Y, Fan Z. Printable fabrication of nanocoral‐structured electrodes for high‐performance flexible and planar supercapacitor with artistic design. *Adv. Mater.* **29**, 1701736 (2017).
69. Bräuniger Y, Lochmann S, Grothe J, Hantusch M, Kaskel S. Piezoelectric inkjet printing of nanoporous carbons for micro-supercapacitor devices. *ACS Appl. Energy Mater.* **4**, 1560-1567 (2021).
70. Jiang Q, Kurra N, Alhabeb M, Gogotsi Y, Alshareef HN. All Pseudocapacitive MXene‐RuO_2 Asymmetric Supercapacitors. *Adv. Energy Mater.* **8**, 1703043 (2018).

71. Zhang CF, *et al.* Additive-free MXene inks and direct printing of micro-supercapacitors. *Nat. Commun.* **10**, 1795 (2019).
72. Wang Y, *et al.* Direct Graphene-Carbon Nanotube Composite Ink Writing All-Solid-State Flexible Microsupercapacitors with High Areal Energy Density. *Adv. Funct. Mater.* **30**, 1907284 (2020).
73. Yang W, *et al.* Carbon‐MEMS‐based alternating stacked MoS_2@rGO‐CNT micro‐supercapacitor with high capacitance and energy density. *Small* **13**, 1700639 (2017).
74. Bellani S, *et al.* Scalable Production of Graphene Inks via Wet-jet Milling Exfoliation for Screen-Printed Micro-Supercapacitors. *Adv. Funct. Mater.* **29**, 1807659 (2019).
75. Yan Y, *et al.* All-in-one asymmetric micro-supercapacitor with Negative Poisson's ratio structure based on versatile electrospun nanofibers. *Chem. Eng. J.* **433**, 133580 (2022).
76. Li J, *et al.* Cladding nanostructured AgNWs-MoS_2 electrode material for high-rate and long-life transparent in-plane micro-supercapacitor. *Energy Storage Mater.* **16**, 212-219 (2019).
77. Liu J, Khanam Z, Ahmed S, Wang T, Wang H, Song S. Flexible antifreeze zn-ion hybrid supercapacitor based on gel electrolyte with graphene electrodes. *ACS Appl. Mater. Interfaces* **13**, 16454-16468 (2021).
78. Yuan C, Zhang X, Wu Q, Gao B. Effect of temperature on the hybrid supercapacitor based on NiO and activated carbon with alkaline polymer gel electrolyte. *Solid State Ionics* **177**, 1237-1242 (2006).
79. Wang M, *et al.* Flexible and low temperature resistant semi-IPN network gel polymer electrolyte membrane and its application in supercapacitor. *Journal of Membrane Science* **597**, 117740 (2020).
80. Huang J, *et al.* Self-powered integrated system of a strain sensor and flexible all-solid-state supercapacitor by using a high performance ionic organohydrogel. *Mater. Horiz.* **7**, 2085-2096 (2020).
81. Liu Y, *et al.* Flexible supercapacitors with high capacitance retention at temperatures from −20 to 100 °C based on DMSO-doped polymer hydrogel electrolytes. *J. Mater. Chem. A* **9**, 12051-12059 (2021).
82. Liu J, Khanam Z, Ahmed S, Wang H, Wang T, Song S. A study of low-temperature solid-state supercapacitors based on Al-ion conducting polymer electrolyte and graphene electrodes. *J. Power Sources* **488**, 229461 (2021).
