# Supplementary material for: 4D printing of MXene hydrogels for high-efficiency pseudocapacitive energy storage
Source: Nat Commun. 2022 Nov 12;13:6884. doi: 10.1038/s41467-022-34583-0 (PMC9653467; doi:10.1038/s41467-022-34583-0)
Supplement: Supplementary file 3 — Description of Additional Supplementary Files [file 41467_2022_34583_MOESM3_ESM.pdf]

Supplementary Movie 1. Printing of microlattice on glass slide using $Ti_3C_2T_x$ ink.

Supplementary Movie 2. Printing of rectangular hollow prism on glass slide using $Ti_3C_2T_x$ ink.

Supplementary Movie 3. Printing of Chinese knot on cloth using Nb_2CT_x ink.

Supplementary Movie 4. Printing of “CRANN” logo on PET film using Nb_2CT_x ink.

Supplementary Movie 5. Printing of micro-supercapacitor units on PET film using $Mo_2Ti_2C_3T_x$ ink.

Supplementary Movie 6. Shaking of 4D-printed $Ti_3C_2T_x$ hydrogels in DI water.

Supplementary Movie 7. Shaking of 3D-printed $Ti_3C_2T_x$ sol in DI water.
